# Go Figure: Transparency in neuroscience images preserves context and clarifies interpretation

**Published:** 2025-04-10

**Authors:** Paul A. Taylor, Himanshu Aggarwal, Peter A. Bandettini, Marco Barilari, Molly G. Bright, César Caballero-Gaudes, Vince D. Calhoun, Mallar Chakravarty, Gabriel A. Devenyi, Jennifer W. Evans, Eduardo A. Garza-Villarreal, Jalil Rasgado-Toledo, Rémi Gau, Daniel R. Glen, Rainer Goebel, Javier Gonzalez-Castillo, Omer Faruk Gulban, Yaroslav Halchenko, Daniel A. Handwerker, Taylor Hanayik, Peter D. Lauren, David A. Leopold, Jason P. Lerch, Christian Mathys, Paul McCarthy, Anke McLeod, Amanda Mejia, Stefano Moia, Thomas E. Nichols, Cyril Pernet, Luiz Pessoa, Bettina Pfleiderer, Justin K. Rajendra, Laura D. Reyes, Richard C. Reynolds, Vinai Roopchansingh, Chris Rorden, Brian E. Russ, Benedikt Sundermann, Bertrand Thirion, Salvatore Torrisi, Gang Chen

**Affiliations:** Scientific and Statistical Computing Core, NIMH, NIH, Bethesda, MD, USA; Inria, CEA, Université Paris-Saclay, Palaiseau, 91120, France; Section on Functional Imaging Methods, NIMH, NIH, Bethesda, MD, USA; Crossmodal Perception and Plasticity Lab, Institute of Neuroscience (IoNS) and Institute of Research in Psychology (IPSY), Université Catholique de Louvain, 1348 Louvain-la-Neuve, Belgium; Department of Physical Therapy and Human Movement Sciences, Feinberg School of Medicine, Northwestern University, Chicago, IL, USA; Department of Biomedical Engineering, McCormick School of Engineering and Applied Sciences, Northwestern University, Evanston, IL, USA; Basque Center on Cognition, Brain and Language, San Sebastian-Donostia, Spain; Ikerbasque, Basque Foundation for Science, Bilbao, Spain; Tri-institutional Center for Translational Research in Neuroimaging and Data Science (TReNDS), Georgia State, Georgia Tech, Emory, Atlanta, GA, USA; Cerebral Imaging Centre, Douglas Mental Health University Institute, Montreal, QC, Canada; Department of Psychiatry, McGill University, Montreal, QC, Canada; Department of Biomedical Engineering, McGill University, Montreal, QC, Canada; Cerebral Imaging Centre, Douglas Mental Health University Institute, Montreal, QC, Canada; Department of Psychiatry, McGill University, Montreal, QC, Canada; Experimental Therapeutics and Pathophysiology Branch, NIMH, NIH, Bethesda, MD, USA; Department of Behavioral and Cognitive Neurobiology, Institute of Neurobiology, Universidad Nacional Autónoma de México campus Juriquilla, Querétaro, Mexico; Department of Behavioral and Cognitive Neurobiology, Institute of Neurobiology, Universidad Nacional Autónoma de México campus Juriquilla, Querétaro, Mexico; Inria, CEAUniversité Paris-Saclay, Palaiseau, 91120, France; Scientific and Statistical Computing Core, NIMH, NIH, Bethesda, MD, USA; Department of Cognitive Neuroscience, FPN, Maastricht University, Maastricht, NL; Brain Innovation, Maastricht, NL; Section on Functional Imaging Methods, NIMH, NIH, Bethesda, MD, USA; Basque Center on Cognition, Brain and Language, San Sebastian-Donostia, Spain; Department of Cognitive Neuroscience, FPN, Maastricht University, Maastricht, NL; Brain Innovation, Maastricht, NL; Department of Psychological and Brain Sciences, Dartmouth College, Hanover, NH, USA; Section on Functional Imaging Methods, NIMH, NIH, Bethesda, MD, USA; Wellcome Centre for Integrative Neuroimaging, FMRIB, University of Oxford, Oxford, UK; Scientific and Statistical Computing Core, NIMH, NIH, Bethesda, MD, USA; Systems Neurodevelopment Laboratory, NIMH, NIH, Bethesda, MD, USA; Wellcome Centre for Integrative Neuroimaging, Nuffield Department of Clinical Neurosciences, University of Oxford, Oxford, UK; Department of Medical Biophysics, University of Toronto, Toronto, ON, Canada; Institute of Radiology and Neuroradiology, Evangelisches Krankenhaus Oldenburg, Universitätsmedizin Oldenburg, Oldenburg, Germany; Research Center Neurosensory Science, Carl von Ossietzky Universität Oldenburg, Oldenburg, Germany; Wellcome Centre for Integrative Neuroimaging, FMRIB, Nuffield Department of Clinical Neurosciences, University of Oxford, UK; Department of Radiology and Nuclear Medicine, University Hospital Magdeburg, Otto-Von-Guericke University, Magdeburg, Germany; Department of Statistics, Indiana University, Bloomington, USA; Department of Cognitive Neuroscience, FPN, Maastricht University, Maastricht, NL; Big Data Institute, Li Ka Shing Centre for Health Information and Discovery, Nuffield Department of Population Health, University of Oxford, UK; Wellcome Centre for Integrative Neuroimaging, FMRIB, Nuffield Department of Clinical Neurosciences, University of Oxford, UK; Neurobiology Research Unit, Rigshospitalet, Denmark; Department of Psychology, University of Maryland, College Park, MD, USA; Clinic of Radiology, Medical Faculty, University of Münster, Münster, Germany; Scientific and Statistical Computing Core, NIMH, NIH, Bethesda, MD, USA; Laboratory on Quantitative Medical Imaging, National Institute of Biomedical Imaging and Bioengineering, NIH, MD, USA; Scientific and Statistical Computing Core, NIMH, NIH, Bethesda, MD, USA; Functional MRI Facility, NIMH, NIH, Bethesda, MD, USA; McCausland Center for Brain Imaging, Department of Psychology, University of South Carolina, Columbia, SC 29208, USA; Center for Biomedical Imaging and Neuromodulation, Nathan Kline Institute, 140 Old Orangeburg Road, Orangeburg, NY 10962, USA; Nash Family Department of Neuroscience and Friedman Brain Institute, Icahn School of Medicine at Mount Sinai, One Gustave L. Levy Place, New York, NY 10029, USA; Department of Psychiatry, New York University at Langone, One, 8, Park Ave, New York, NY 10016, USA; Institute of Radiology and Neuroradiology, Evangelisches Krankenhaus Oldenburg, Universitätsmedizin Oldenburg, Oldenburg, Germany; Research Center Neurosensory Science, Carl von Ossietzky Universität Oldenburg, Oldenburg, Germany; Clinic of Radiology, Medical Faculty, University of Münster, Münster, Germany; Université Paris Saclay, France.; University of California, San Francisco, Department of Radiology & Biomedical Imaging, SF CA; San Francisco Veteran Affairs Health Care System, SF CA; Scientific and Statistical Computing Core, NIMH, NIH, Bethesda, MD, USA

## Abstract

Visualizations are vital for communicating scientific results. Historically, neuroimaging figures have only depicted regions that surpass a given statistical threshold. This practice substantially biases interpretation of the results and subsequent meta-analyses, particularly towards non-reproducibility. Here we advocate for a “transparent thresholding” approach that not only highlights statistically significant regions but also includes subthreshold locations, which provide key experimental context. This balances the dual needs of distilling modeling results and enabling informed interpretations for modern neuroimaging. We present four examples that demonstrate the many benefits of transparent thresholding, including: removing ambiguity, decreasing hypersensitivity to non-physiological features, catching potential artifacts, improving cross-study comparisons, reducing non-reproducibility biases, and clarifying interpretations. We also demonstrate the many software packages that implement transparent thresholding, several of which were added or streamlined recently as part of this work. A point-counterpoint discussion addresses issues with thresholding raised in real conversations with researchers in the field. We hope that by showing how transparent thresholding can drastically improve the interpretation (and reproducibility) of neuroimaging findings, more researchers will adopt this method.

## INTRODUCTION

Beyond performing experiments and recording data, a scientist is responsible for synthesizing information, interpreting it within the context of prior knowledge, and communicating it effectively. This curation and distillation involves a careful balance of contextualization and concise communication, without losing meaningful information. Data visualization is itself an important analysis step with key processing choices to be made, though their impact is often overlooked and underappreciated. Here we examine these aspects for results reporting in neuroimaging, where typical datasets are complex and multi-dimensional. We start by describing how the field and its research questions have changed over time, and then discuss how the presentation of results in brain images should similarly evolve. The proposed improvements are straightforward and implementable in a large number of widely used software packages. Many of the presented examples use functional magnetic resonance imaging (FMRI) data, but the concepts and methods of improving results reporting are directly applicable to other imaging modalities.

### Background: historical context

Localization was an early focus of neuroimaging researchers, who broadly conceptualized the brain as a set of discrete regions with well-defined functions to be identified and displayed, in line with empirical procedures from lesion based neuropsychology and early cognitive psychology (e.g., see Savoy, 2001). Unfortunately, neuroimaging signals—particularly FMRI recordings—are noisy, dynamic, and contain smooth patterns whose size and shape is hard to delineate. To combat these initial challenges, neuroscientists turned their attention to standard null hypothesis significance testing as a way to implement a clear filtering mechanism. Within this framework, strict thresholding was a key processing step for localizing a small number of regions of interest and reducing potential false positives (Forman et al., 1995; Nichols and Hayasaka, 2003; Smith and Nichols, 2009). An emphasis was placed on the results having a small number of suprathreshold clusters and rigorous boundaries of statistical significance. Subthreshold regions were interpreted as simply noise or non-neuronal features (negative-signed activations were even filtered in many cases, prior to interest in task-negative networks). Therefore, researchers presented results with strict or “opaque” thresholding: wherever a statistic value was subthreshold, all modeling results—including the effect estimate, *p*-value and statistic itself—are fully hidden from view and withheld from consideration.

However, this approach comes with inherent challenges. The adoption of univariate statistics on interdependent data like voxels and vertices requires statistical adjustment for multiple comparisons to reduce false positives across these thousands of tests. At the same time, this also causes meaningful effects to fall below the substantially decreased statistical power (Cremers et al., 2017; Lohmann et al., 2017; Bacchetti, 2013), causing a “tip of the iceberg effect” (Pang et al., 2023; Noble et al., 2024; Sundermann et al., 2024). Adopting multivariate analyses (e.g. searchlight-based multivariate pattern analysis, MVPA) does not solve this problem, since statistical peaks might not correspond to biologically meaningful locations, but instead to the center of maximally informative neighborhoods (Etzel et al., 2013). Consequently, replication studies using similarly stringent thresholds may yield results that appear strikingly different, potentially causing undue concerns about low reliability and highlighting the limitations of this traditional framework. Even within a single study, relevant results may fall below the strict corrective adjustments (i.e., fall “below the waterline”), biasing the evaluation and interpretation.

Over time, methodological and conceptual changes have also occurred in the field. Modern neuroimaging presents a more intricate and nuanced picture of brain activity. Network-based and connectomic studies have become much more common, shifting to a paradigm in which most functions involve the interaction of many parts of the brain to varying degrees (e.g., Calhoun et al, 2001; Calhoun et al., 2002; Greicius et al., 2003; Damoiseaux et al., 2006; Hagmann et al., 2008; Fair et al., 2009; Smith et al., 2009; Biswal et al., 2010; Van Essen et al., 2013; Pessoa, 2014). While regions with high statistical strength might still be particularly important, they do not simply indicate small regions turning on/off in isolation. Responses modulate, and importance also lies with other parts of their (or other) networks that might have weaker effects (e.g., see Noble et al. 2024). That is, even when focused on “significant clusters,” the subthreshold results across the rest of the brain will still provide necessary context for understanding their role and for having a more complete picture of how the brain is behaving. Gonzalez-Castillo et al. (2012)’s deep scanning study further showed brain activation at many scales and changing extents of regional activation with increased data. Recent approaches of modeling eigenmodes of brainwide function have reinforced the importance of visualizing nonlocal and subthreshold effects (Pang et al., 2023).

As a result, the idea of an exactly zero response in any gray matter seems unlikely. Treating it as such—which is what standard opaque thresholding effectively does—creates statistical issues (Cremers et al., 2017). Even if zero effects existed, concluding that subthreshold test statistics imply zero effect amounts to confirmation of the null hypothesis, which goes against the principles of hypothesis testing. In the view of modern neuroimaging, opaque thresholding also wastes meaningful information, since responses and effects are not simply localizable in an on/off manner, as responses occupy a continuous spectrum (Chen et al., 2022). This is one reason that clinical practitioners often include fully unthresholded images in their assessments, to see more context and reduce false negatives (Voets et al., 2025). In general, the results of a neuroimaging study should account for the non-dichotomous nature of data by including subthreshold information, both when the study authors are interpreting it and when readers are engaging with their work.

### Reporting results: the past and the future

The issue of how figures are made might seem like merely a stylistic choice, but it is central to how scientists evaluate and interpret results, how readers assess them, and how meta-analyses compare them. Data visualization is an analysis step, and thresholding data is one of the final processing choices researchers make in a study. It has important consequences for evaluating and understanding results.

The current standard practice of opaque thresholding is rooted in the assumptions of the earliest neuroimaging studies. It has remained largely unchanged and, as a consequence, so have the basic figures and representations of results in neuroimaging studies, even though our understanding of brain function has grown in many ways. Previous work has noted problems with opaque thresholding (Allen et al., 2012; Chen et al., 2022; Taylor et al., 2023; Sundermann et al., 2024). Motivated by this, here we identify and focus on three ways that opaque thresholding negatively impacts the fundamental interpretations and comparisons of neuroimaging results:

1. **Unrealistic biology:** Opaque thresholding treats all subthreshold regions as if they had zero effect rather than simply statistically weaker observations. This creates an unrealistic ON/OFF picture of localized effects, and is not consistent with the current understanding of brain functioning.
2. **Ambiguity:** Brain regions are generally part of overlapping networks, rather than purely isolated and independent. Opaque thresholding removes the context of any results: how quickly effects drop off spatially, what network(s) a cluster is involved with, etc. Other parts of the brain might have higher uncertainty but they still contain useful context, such as evidence for the full range of effects, wider network interpretations, and comparisons.
3. **Bias:** The thresholding of a continuous brain effect or related metric mathematically introduces biases and hypersensitivity to small (often arbitrary) differences, such as between effects minimally below and above the threshold. These will negatively impact within-study evaluation and cross-study reproducibility.

As a consequence, opaque thresholding undermines the content of the results themselves. It compromises the ability to make holistic and accurate interpretations by study authors, readers, and meta-analyses at the most fundamental levels.

To improve neuroimaging visualization, Allen et al. (2012) proposed transparent thresholding as an effective way to include *both* suprathreshold *and* subthreshold information in reported results. This is a simple, meaningful, and straightforward solution whereby suprathreshold regions are highlighted in an image by being opaque and outlined, while subthreshold results are also included by using an opacity that decreases with their absolute statistical value. This balanced approach allows for having a concise summary of regions with the strongest effects (the same suprathreshold regions in existing figures), together with a graded assessment of activity across the rest of the brain (reporting information across the “missing majority” of the data that had been gathered and analyzed). By shifting to transparent thresholding, the data modeling and results are more accurately represented, *no information is lost*, and meaningful evidence is gained.

To date, this visualization approach has not been widely adopted in the field, but it has been applied effectively in several neuroimaging studies (a sample of these are provided in Table S1 of the Supplements). Its utility in providing more complete evaluations of results has also been shown in direct comparisons with standard, opaque thresholding. For example, Taylor et al. (2023) used transparent thresholding to reveal a previously underappreciated degree of consistency and reproducibility in the FMRI results of the Neuroimaging Analysis Replication and Prediction Study (NARPS; Botvinik-Nezer et al., 2020).

Here, we provide four examples that demonstrate the many benefits of retaining context in results. Each highlights an important aspect of reporting and interpreting results that is notably improved with transparent thresholding, namely: improving the understanding of a study, reducing hypersensitivity to arbitrary features (sample size, many processing choices and more), avoiding problematically selective reporting, and enhancing meta-analyses. In the later examples, we also argue that, relatedly, effect estimates from modeling should also be visualized whenever available. These provide an additional source of important information in both figures and comparisons. In the Discussion, we address several considerations and concerns that researchers in the field have raised about transparent thresholding. Changes to conventions often face hesitation, and these points are important to consider. But the scientific costs of using opaque thresholding are demonstrably high, and the benefits of showing context with transparent thresholding are both clear and abundant.

We note that one barrier to adopting a new approach is its availability to researchers. We highlight that transparent thresholding is now widely available across a large number of neuroimaging software packages. While a small number of toolboxes have previously included transparent thresholding, several others have been recently implemented or significantly streamlined this approach during this project. These tools are listed (with example images) in the Discussion, and further details are contained in the Supplements.

## RESULTS

We present four examples using real data that illustrate the importance of showing context in brain images. These beneficial features include: removing ambiguity in interpreting the data (Ex. 1); accurately comparing data and assessing differences, both visually and in more formal meta-analyses (Ex. 2); avoiding hypersensitivity to non-physiological features, such as sample size (Ex. 3); and reinforcing robust and accurate interpretations (Ex. 4).

### Example 1: context to reduce ambiguity

**Figure 1.**
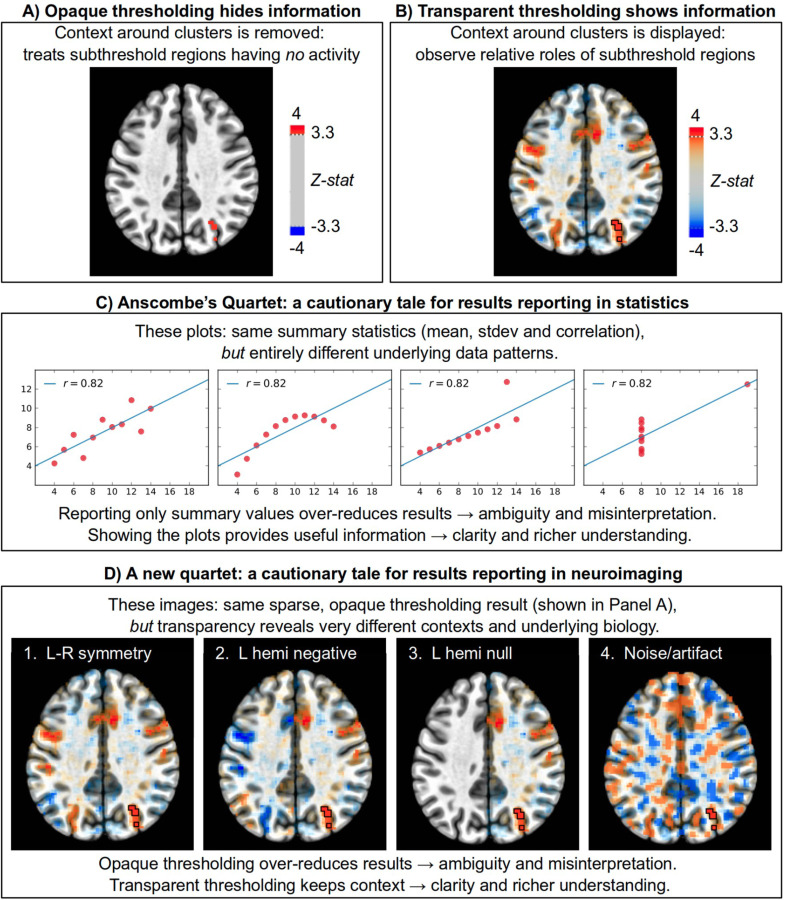
Results reporting examples, showing a single slice of task-based FMRI data (see Chen, Pine, et al., 2022). Each neuroimaging panel shows the same axial slice in MNI template space at z = 36S (image left = subject left), with thresholding is applied at voxelwise p = 0.001 and cluster size = 40 voxels (FWE = 5%). The data used for both overlay coloration and thresholding are the Z-score statistics. Panel A displays FMRI results using conventional strict (or opaque) thresholding, and shows one cluster in the right intraparietal sulcus. Panel B displays the same results with transparent thresholding (suprathreshold regions are opaque and outlined; subthreshold regions fade as the statistic decreases), revealing relevant context in the subthreshold regions that are hidden in A. Panel C shows a classic example from Anscombe (1973) of the risks of over-reducing data, here for a simple scatterplot. Panel D shows how the same considerations apply to neuroimaging: each dataset would have very different interpretations and biological implications, which can be appreciated with transparent thresholding (same colorbar as B), but when using opaque thresholding that context is lost and each slice reduces to the same image (that of panel A). Only by displaying the more full context with subthreshold visualization can the degeneracy be broken and results more accurately understood. Opaque thresholding removes context and can often lead to a misinterpretation of results.

Fig. 1A shows group results from a set of Flanker task-based FMRI data (see Chen, Pine, et al. 2022) presented in the conventional style. In this figure, data processing includes opaque thresholding at a family-wise error (FWE) rate of 5% (via voxelwise *p* = 0.001 and cluster-correction N = 40 voxels). The displayed results only contain the lobes of a single cluster appearing in the right intraparietal sulcus. Since opaque thresholding is applied, no other information is conveyed for this slice than the fact that the absolute value of the statistic at every non-cluster location was subthreshold. Some potentially interesting clusters might be just one voxel below the estimated cut-off, but they remain entirely hidden. The reader can only interpret the biological implications of this study from this sparse statistical information alone: thus, only a single region appears to show significant response, and the activity seems fully lateralized in the right intraparietal sulcus.

The scale of information loss can be appreciated by viewing Fig. 1B, where the thresholding has been applied transparently to retain context in the same data. This reveals a brainwide set of nontrivial responses, many of which seem biologically relevant despite being statistically subthreshold. Note that the opacity fades quadratically with decreasing statistic value, so the most notable “extra” results are still near the threshold level (compare the colorbars in Figs. 1A-B). Seeing this context assists in interpreting the most significant regions, which are still highlighted with full opacity and outlines. It also reduces possible misinterpretation. For example, the richer context of the modeling results implies that the “lone lateralized cluster” actually has much stronger left-right symmetry than judged from Fig. 1A (though still with a larger response on the right). The arbitrary influence of opaque thresholding on laterality measures has been particularly noted in clinical contexts (Ruff et al., 2008; Seghier, 2008; Suarez et al., 2009), and though not yet widely adopted, transparent thresholding would greatly improve evaluations. We also see that opaque thresholding has over-reduced the results of the whole-brain modeling, while Fig. 1B contains a more accurate representation of the full study evidence and invites further research to explore the involvement of network components (several of which also appear focal and lateralized).

The interpretational consequences of over-reducing results are not just a quirk of FMRI, and cases from other fields provide useful lessons. For instance, in the classic example of Anscombe’s Quartet (Anscombe, 1973), four scatterplots show data that have the exact same summary statistics and correlation value, but with very different underlying patterns (see Fig. 1C). If the results of the correlation analysis are only reported as summary statistics, important information is lost and one is highly likely to make an entirely incorrect inference about the data itself. Only by keeping the contextual information of the plot can the ambiguity of the summary values be resolved. Acknowledgment of these issues has led researchers in many areas to take action in improving their statistical reporting: they show scatterplots of data (not just fit values); they use violin plots and raincloud plots to detail distributions; and more.

Identical reasoning further emphasizes the importance of using transparent thresholding to retain context in neuroimaging reports. Fig. 1D shows images of four sets of possible results, related to the Flanker task in Fig. 1A. Transparent thresholding allows us to see that there are major differences among them, but with opaque thresholding they each reduce exactly to Fig. 1A and are indistinguishable. That is, opaque thresholding inherently produces problematic ambiguity because the interpretation of the statistically significant cluster would be dramatically different across the four cases, with each image representing a very different biological implication: 1) fairly symmetric left-right activation (actual response); 2) *anti*-symmetric left-right activation; 3) strongly right-lateralized response; 4) likely a noise- or artifact-driven outcome rather than a task-related physiological one. From the loss of context with opaque thresholding, there is little choice but to apply Occam’s Razor and interpret the results as pointing toward strong or absolute laterality (something like Image #3 in Fig. 1D), which the fuller context does not support. By thresholding transparently, one observes a more complete representation of the results while still having the most significant regions highlighted.

Seeing results beyond the tip of the iceberg also improves localization, by revealing the spatial drop-off of the response (here, in terms of statistical value, but below we show how the effect evidence can be presented). This further provides useful information about the properties of the data itself such as its spatial smoothness, which reflects both acquisition and processing. Interestingly, spatial smoothness was highlighted as a major factor for varied outcomes in the NARPS project (Botvinik-Nezer et al., 2020), so having this information directly available in primary figures may be particularly useful for cross-study comparisons. Finally, subthreshold visualizations can help distinguish among potential underlying features, such as motion versus respiratory effects, additionally benefitting quality control and assurance efforts.

### Example 2: context to improve cross-study comparison and meta-analysis

Retaining context is particularly important for comparing datasets and performing meta-analyses. The NARPS project gathered results from approximately 70 teams who had processed the same task-based FMRI data collection independently and reported on specific hypotheses. In their primary comparison of FMRI results, the NARPS authors assessed the similarity of binarized yes/no responses to nine region-specific hypotheses, based on opaquely thresholded statistical results. They reported observing a “substantial variability in reported binary results, with high levels of disagreement across teams on a majority of tested hypotheses.” They also noted that the similarity of teams’ opaquely thresholded maps, as measured by cluster overlap, was low. However, when they compared the *unthresholded* results, they found “a large cluster of teams had statistical maps that were strongly positively correlated with one another” and that “analyses of the underlying statistical parametric maps on which the hypothesis tests were based revealed greater consistency than expected from those inferences.” That is, comparisons on results processed with standard thresholding showed relative disagreement, while those without thresholding showed a large amount of agreement. The second message has tended to be greatly underemphasized, and this widely cited paper has overwhelmingly been referenced simply as evidence of *high* variability across processing pipelines. Instead, it should be viewed as a demonstration of the influence thresholding has as a processing step and an important warning of the biases of opaque thresholding, which preferentially tilt study comparisons towards non-reproducibility.

**Figure 2.**
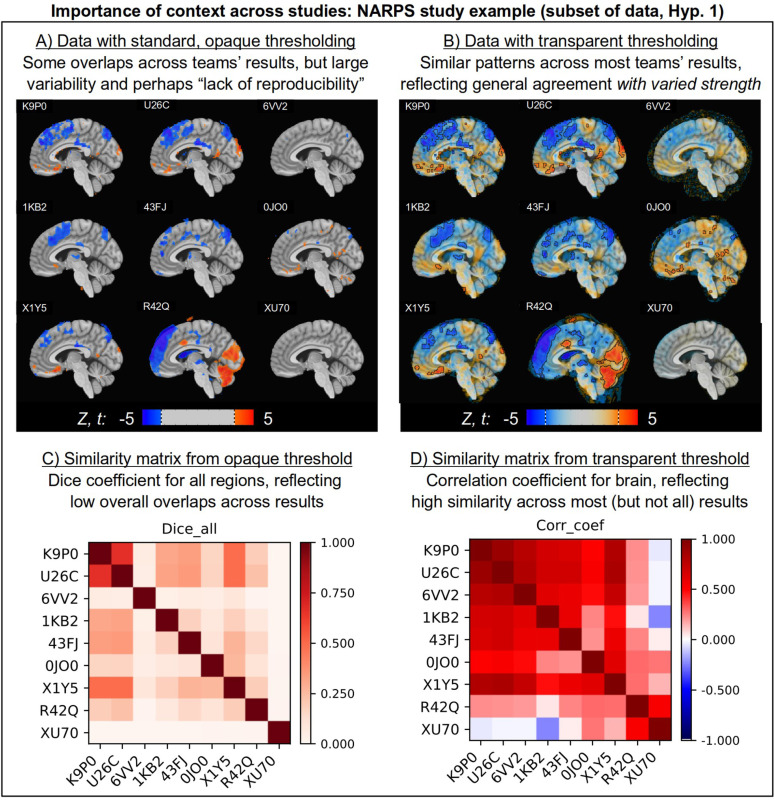
Visualizing results from 9 teams who participated in NARPS (Botvinik-Nezer et al., 2020); team IDs are shown in each panel. Panels A-B show Z and t statistic maps in the same sagittal slice of MNI space and thresholded at |Z| or |t| = 3. Panel A shows the results with opaque thresholding (in line with the study’s primary meta-analysis), which suggest high variability, inconsistency and disagreement across teams. Panel C shows the corresponding similarity matrix (using Dice coefficients for the binarized cluster maps), which quantifies the generally poor agreement. Panel B shows the same data with transparent thresholding (in line with the study’s second meta-analysis), where it becomes apparent that the results actually agree strongly for most subjects, but with varied strength. Panel D shows the corresponding similarity matrix (using Pearson correlation for the continuous statistic maps), showing the typically higher similarity. Transparent thresholding does not uniformly increase similarity, but allows for clearer interpretation of real differences (e.g., bottom right image). Opaque thresholding biases towards dissimilarity (e.g., 3rd column, top and middle). See Taylor et al. (2023) for similar comparisons across the full set of NARPS teams and hypotheses, where the same patterns hold.

We can see how the choice of thresholding explains the opposing meta-analytic results—high variability when thresholding vs surprising similarity without it—by visualizing a representative set of teams’ results. Fig. 2A shows nine teams’ results from analyzing the same FMRI dataset for NARPS Hypotheses 1, and applying opaque thresholding at *|Z|* or |*t*| = 3 (equivalent quantities due to the degree of freedom count; see Supplements). While a few of the teams share somewhat similar suprathreshold clusters, several contain almost no results (3rd column) or sparse regions, yielding an impression of high variability across the teams, consistent with the NARPS authors’ primary meta-analysis findings. However, switching to transparent thresholding (Fig. 2B) immediately reveals that the statistical patterns are actually quite similar across the majority of teams, just with differing magnitudes. That is, the spatial patterns have high correlation, consistent with the secondary meta-analytic findings in NARPS. These observations are quantified and summarized in the similarity matrices shown in Fig. 2C and 2D, which are again consistent with NARPS’s primary and secondary meta-analyses, respectively. Taylor et al. (2023) showed how these patterns of predominantly high similarity (with a small number of outliers) persist across the full set of teams’ results and all hypotheses, when applying transparent thresholding.

This example demonstrates how having the full context in the images is key to understanding the full scope of results—particularly the *kind* of variability that is present.^1^ The dominant variability across the NARPS teams’ results is actually that of the *magnitude* of the statistics, rather than of the sign or spatial pattern of statistical maps. The opaquely thresholded maps cannot distinguish between these kinds of variability below an elevated cut-off point, and hence they bias the interpretation towards one of simply high variability and “lack of reproducibility.” Seeing the full context as part of the comparison helps to reduce and resolve this bias.

Note that while opaque thresholding biases comparisons towards non-reproducibility, using transparent thresholding to retain context does *not* necessarily increase similarity. It merely allows for better informed comparisons. Consider the third column in each of Fig. 2A and 2B: while transparent thresholding reveals meaningful statistical patterns in the upper two images, it also shows that the bottom image values are uniformly quite low and negligible by comparison. This is another case of resolving ambiguity, as described in Fig. 1: what ostensibly appeared to be three examples of the same thing under opaque thresholding were actually revealed to be distinct by using transparency. Seeing more context in the bottom-middle image reveals that its results have much lower similarity to the others above it, which is also reflected in its much lower correlation value (Fig. 2D). In all cases, these more informative observations can lead to a more impartial assessment of a particular team’s results, and encourage a helpful examination of the corresponding preprocessing and analytic choices.

Thus, while there is variability in the NARPS teams’ processing and results, two different meta-analysis approaches provide very different assessments of it both visually and quantitatively. The one associated with opaque thresholding (which hides useful information, leaves ambiguity, and biases the comparison towards dissimilarity) suggests high variability. In contrast, the one associated with transparent thresholding (which leaves context, provides more modeling evidence, and represents a more complete picture of the data) suggests widespread similarity with varied magnitude. That is, *rather than being an inherent feature of the teams’ results, the outcome of high variability is primarily due to the processing choice of applying opaque thresholding prior to comparison, which introduces a strong bias.* Instead, retaining context in the datasets improves both the mechanics and interpretation of the meta-analysis.^2^

### Example 3: context to reduce hypersensitivity and instability of results

In this example, we contrast the hypersensitivity of opaquely thresholded results to non-physiological features with the relative stability of transparently thresholded ones. Aspects of this have been shown above in the discussion of the NARPS data, but here we demonstrate this point even more directly with a simple case of varying the number of subjects in a study.

**Figure 3.**
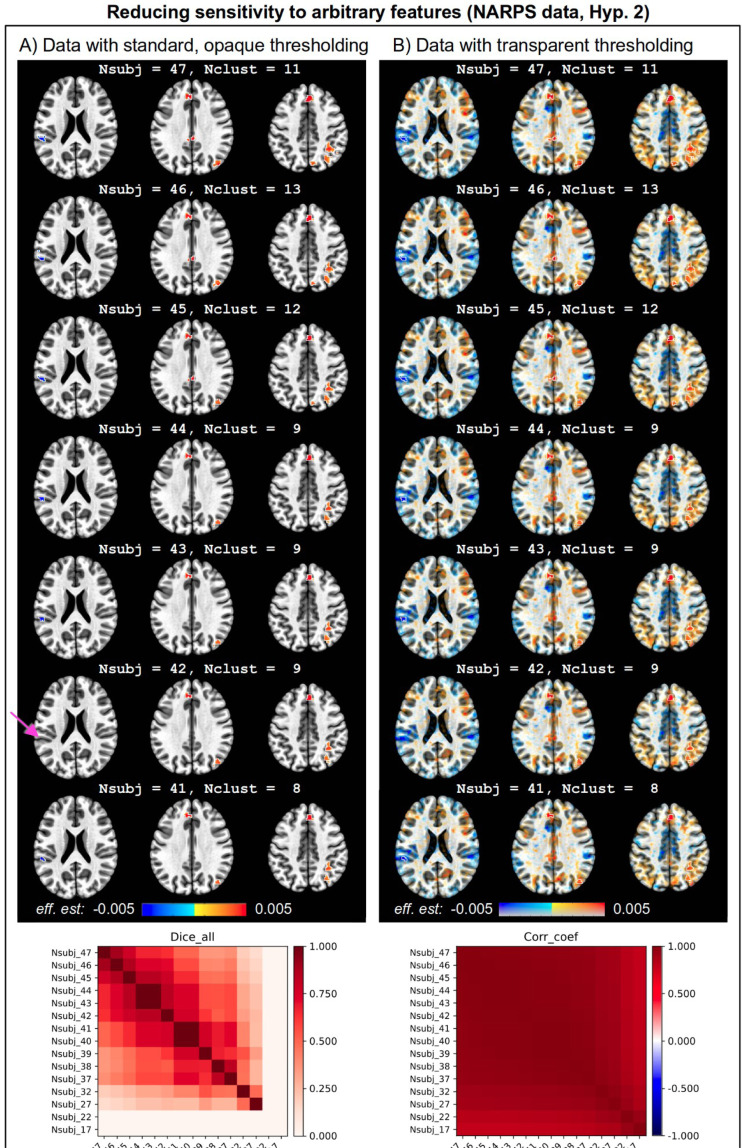
Each panel shows the same axial slices in MNI template space at z = 21S, 32S, 43S (image left = subject left) from NARPS data, Hyp. 2 and 4. The overlay values are effect estimates, in units of BOLD% signal change per dollar for this gambling task, and statistic values were used for thresholding (voxelwise p = 0.001; cluster-level FWE = 5%). All suprathreshold clusters are highlighted with white outlines, for visibility. The top row shows the full group number of subjects (Nsubj), and subsequent rows show results with 1 subject removed. Changes in cluster count (Nclust) are noted for each row. Changes in cluster results —in terms of both coverage and number—are more apparent in Panel A, where opaque thresholding is used. The changes are not simply convergent or monotonic. The magenta arrow highlights a cluster in the left inferior parietal lobule which disappears and reappears with varying Nsubj. The results with transparent thresholding in Panel B are less sensitive to Nsubj changes and also provide useful context. For example, the region highlighted with the magenta arrow appears to have left-right symmetry in negative BOLD response; this information is missed with opaque thresholding. The bottom of each column shows a similarity matrix for each thresholding style (as in Fig. 2), for an extended set of Nsubj. These reflect the striking sensitivity of opaque thresholding (Dice_all, left) with the more stable transparent thresholding (Corr_coef, right).

Fig. 3 displays the results of a standard one-group analysis with the NARPS data (for Hyp. 2), using a two-sided *t*-test with cluster-based FWE = 5%. Clusters are outlined in white for visibility. The top row contains the results from analyzing the full set of 47 subjects used for group analysis after processing and quality control. Subsequent rows show results if the group size had been reduced by just one subject (arbitrarily chosen by order of subject ID). Fig. 3A shows the results with opaque thresholding applied—both the coverage and number of clusters change notably from row to row. In one case, removing just one subject *decreased* the number of clusters by 25%, and in another case it *increased* the count by 18%. The changes are not simply monotonic or convergent, and one cluster disappears and then reappears (in the left inferior parietal lobule; magenta arrow). A similarity matrix of the clusters (bottom row) shows the amount of variability across an extended set of group sizes (down to 17). Clearly, any interpretation of results with this opaque thresholding will be quite sensitive to group size.

Fig. 3B displays the same results using transparent thresholding. The images are considerably more consistent across the small group size changes, reflecting less sensitivity to the arbitrary differences (e.g., quality control criteria, censoring thresholds, subject motion values, etc.). Note that the changes in cluster count are still known and still vary in the same way, but the contextualized maps allow for the reader’s evaluation to remain appropriately consistent and stable. The similarity matrix for these results (Fig. 3D) shows uniformly quite high values even down to the smallest group size. Beyond stability, additional benefits of transparency include having knowledge of the context itself. For example, the area highlighted with the magenta arrow appears to have notable left-right symmetry, which would be unknown in the opaque thresholding case.

Study results are always produced and examined in the context of prior information. The hypersensitivity of opaquely thresholded clusters makes them difficult to rely on for robust comparisons to other papers and even for meaningful evaluation of a study’s hypotheses. Opaque thresholding creates the dilemma of determining, for example, the appropriate final number of subjects from which to obtain the “correct” set of clusters. Moreover, it creates a potential incentive for *p*-hacking (Wichert et al., 2016): tweaking and selecting such parameters in a way that might reject the null hypothesis or match more closely with prior work. As shown here, transparent thresholding greatly reduces such temptations, as presented results are more stable and can still be discussed easily, even if just below threshold.

### Example 4: using figures and context to avoid misinterpretation

As a final example of the importance of keeping context in figures to clearly communicate science results, we look back at one of the most widely known studies in FMRI, the “dead salmon study” (Bennett et al., 2009). This study had a single, simple message: when performing massively univariate voxelwise analyses in brain studies, one should adjust for multiple comparisons in some way, such as applying familywise error (FWE) or false discovery rate (FDR) adjustment. This message is clearly stated in the title of the paper, and it is repeatedly restated throughout the abstract and main text. However, the work has still been consistently misreferenced as showing that FMRI is unreliably susceptible to false results (predominantly outside the field) and even frequently misquoted within the field itself.

**Figure 4.**
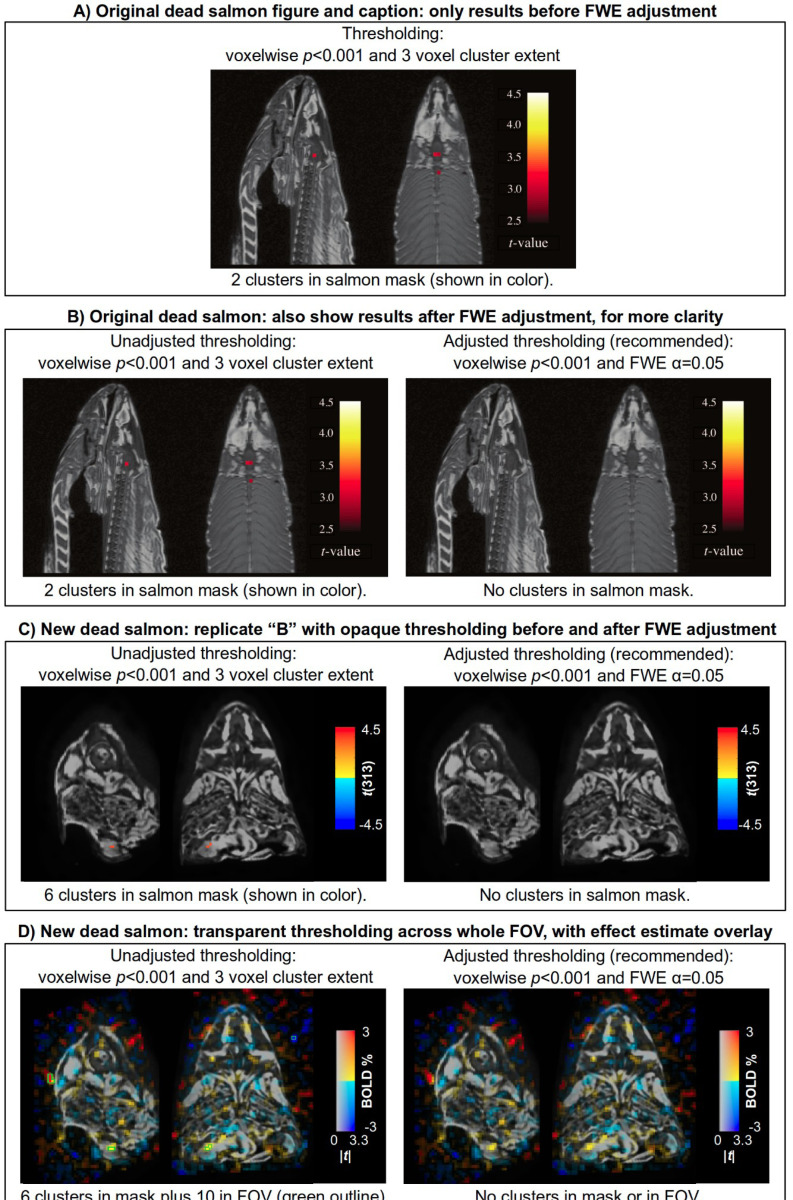
Panel A shows the lone figure from the famous “dead salmon study” (Bennett et al., 2009; with permission of the authors). The figure is opaquely thresholded and only shows results before the recommended multiple comparisons adjustment; by not including the after image, many readers have misinterpreted the overall study message, even though it is clearly repeated throughout the text. Panel B shows a simple improvement to make the figure’s message clearer and reduce the likelihood of misinterpretation, by including both before- and after-adjustment images. Panel C shows the new validation salmon in the same manner as Panel B, replicating the original results with opaque thresholding. Panel D shows how more complete context can be added to further reduce risks of misinterpretation by thresholding transparently (suprathreshold regions outlined in green), displaying the effect estimate in units of BOLD % signal change as overlay colors, and even showing results outside the subject anatomy. This extra information would provide valuable evidence that any cluster that might survive here—which is possible even when including multiple comparisons adjustment—is likely noise due to the background pattern, high noise floor and likely low effect estimate value.

The part of the paper that unfortunately leaves room for misinterpretation is its lone figure. In practice, figures often leave stronger impressions of results with readers than text. The famous image is reproduced here in Fig. 4A, along with relevant descriptive information summarized from the original caption. The image shows opaquely thresholded results^3^ *before* adjusting for multiple comparisons, but not *after* doing so. The authors did perform multiple comparisons adjustment—indeed, that is the analysis step they are promoting—but they simply stated its outcome of “no clusters” *in the text only*. This latter part appears to be ignored by readers relatively frequently, leaving a false impression from the lone, pre-adjustment figure.

One clarifying step would be to include both the “before” and “after” cases in the image, such as in Fig. 4B. The figure’s primary message is now more clearly in line with the methodology and the authors’ intended purpose.

But the results reporting could still be improved further by using transparent thresholding. This is shown in Fig. 4D for a different fish,^4^ scanned during a standard flashing checkerboard stimulus paradigm (see Supplements for details). We again include both the “before” and “after” cases of statistical adjustment, which match those of the original dead salmon (Fig. 4C shows the new validation salmon results with opaque thresholding). By including subthreshold modeling results in the images in Fig. 4D, it is immediately apparent that the overall pattern is obviously quite noisy and unrelated to structure. This context is useful because it is quite possible that a noisy cluster *could still survive* all of the statistical adjustment and thresholding processes. Seeing the noisy subthreshold context would then provide useful (if not necessary) evidence that any such cluster was likely to be noise-related rather than a function-related cluster (similar to Image 4 in Fig. 1D).

In addition to transparent thresholding, Fig. 4D includes additional useful features. First, It includes data from the whole field of view, so one can judge the pattern of results inside the brain with respect to the “noise floor” background (Taylor et al., 2023). Second, the overlay is the effect estimate dataset (BOLD percent signal change), rather than just the statistics, which are still used for thresholding (Chen et al., 2017). In this way, the reader can appreciate that the effect estimate values are quite low within the subject, even though the statistic values there are relatively high in many places. These features apply even beyond applying transparent thresholds to “before” and “after” images, and should be used whenever possible. For example, seeing the very small effect size of a cluster that happened to survive would provide further useful evidence as to its true, noisy nature; this would apply to the cluster in the lower part of the image in Fig. 4C, if it had been just slightly larger. Thresholding transparently, displaying the effect estimate, and including the background FOV provide useful contextual information to solidify and clarify the interpretation.

## DISCUSSION

Neuroimaging authors need to supply enough details for the analysis to be understood and replicable (e.g., Maumet et al., 2016; Nichols et al., 2017). They also need to balance presenting “digestible” results with retaining meaningful information content (Chen, Taylor, et al. 2022). The question of how much data to present and in what form is important, and we can turn again to Anscombe (1973), who commented on the value of graphs to provide useful contextual information beyond just summary statistics:

Graphs can have various purposes, such as: (i) to help us perceive and appreciate some broad features of the data, (ii) to let us look behind those broad features and see what else is there. Most kinds of statistical calculation rest on assumptions about the behavior of the data. Those assumptions may be false, and then the calculations may be misleading. We ought always to try to check whether the assumptions are reasonably correct; and if they are wrong we ought to be able to perceive in what ways they are wrong. Graphs are very valuable for these purposes.

In a near-exact parallel, we believe that transparent thresholding provides important value for understanding and evaluating results in neuroimaging. Many assumptions associated with opaque thresholding are inconsistent with the data, thereby increasing odds of misinterpretation and biases. In contrast, retaining context provides the benefits of both “appreciating broad features of the data” and letting readers “see what else is there.”

The four examples presented here illustrate these points and other benefits of retaining context in neuroimaging results. The primary benefit is to provide a clearer, deeper, and more accurate understanding of the data, for both authors and readers. This is the ultimate goal of a scientific experiment. Results reporting should prioritize having comprehensive information over artificial dichotomization. Removing context with opaque thresholding inserts a large amount of ambiguity and bias into results, tilting the scales towards misinterpretation.

The benefits of transparent thresholding—and costs of opaque thresholding—apply across modality and species. Whether analyzing FMRI, diffusion weighted imaging (DWI) or PET (Bishay et al., 2024) data in humans, macaques (Russ and Leopold, 2015) or rodents, showing subthreshold context improves interpretability. It can also be applied equivalently to voxelwise or ROI-based analyses (Chen et al., 2020; Taylor et al., 2023), as well as to both volumetric and surface-based analyses (Sava-Segal et al., 2024; Freund et al., 2025).

The issue of thresholding images touches at the core of the scientific endeavor, particularly for neuroimaging. An individual study rarely provides a definitive answer. Instead, empirical science is an iterative process that builds upon cumulative evidence from multiple studies. Using opaque thresholds treats an individual study as a standalone decision-making tool, which misrepresents the essence of scientific inquiry. In contrast, transparent thresholding assists that process by presenting results as widely informative evidence rather than as a narrowly defined “answer”. It is also directly in line with larger mathematical recommendations about improving the use and interpretation of statistics and *p*-values across broad scientific disciplines, which include having an “interpretation of results in context” and making “complete reporting” (Wasserstein and Lazar, 2016). By focusing on the broader context and appropriately including results that simply have higher uncertainty, researchers can better align with the collaborative and progressive nature of empirical investigation.

**Figure 5.**
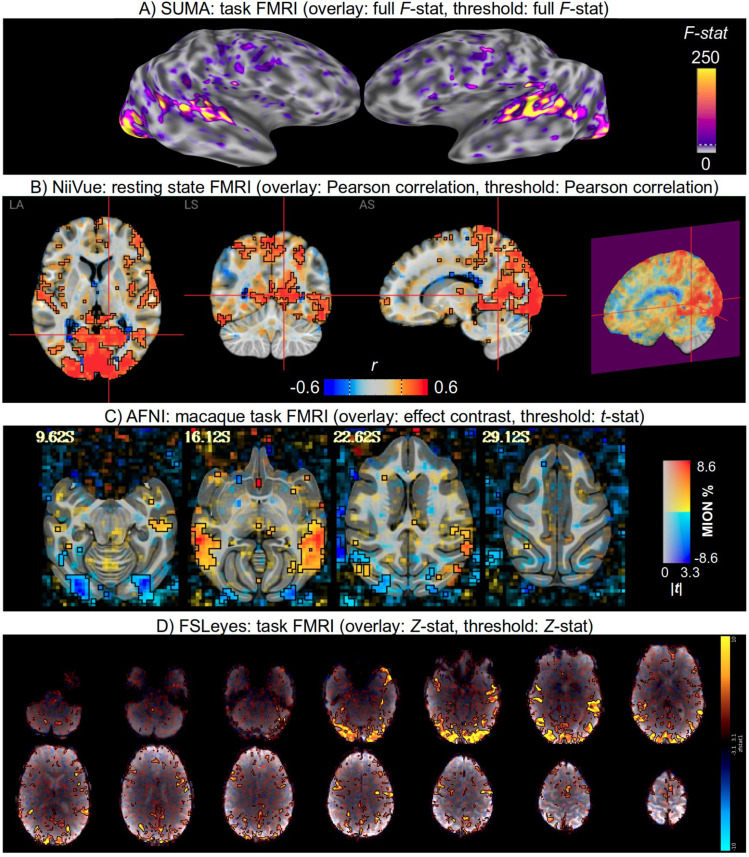
Example images of transparent thresholding from various software implementations (and see Figs. 6 and 7 for more examples). Descriptions of the data and software usage are provided in the Supplements.

**Figure 6.**
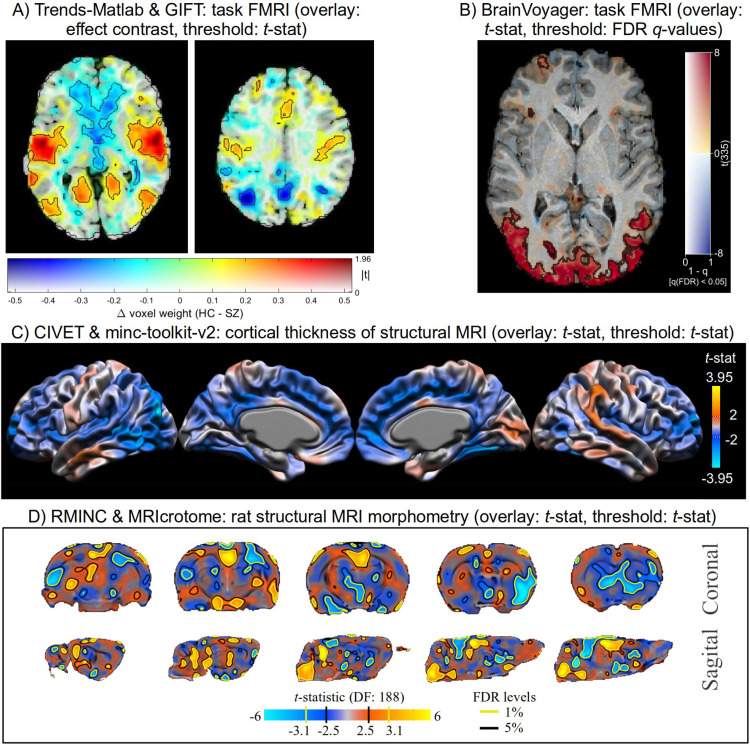
Example images of transparent thresholding from various software implementations (and see Figs. 5 and 7 for more examples). Descriptions of the data and software usage are provided in the Supplements.

**Figure 7.**
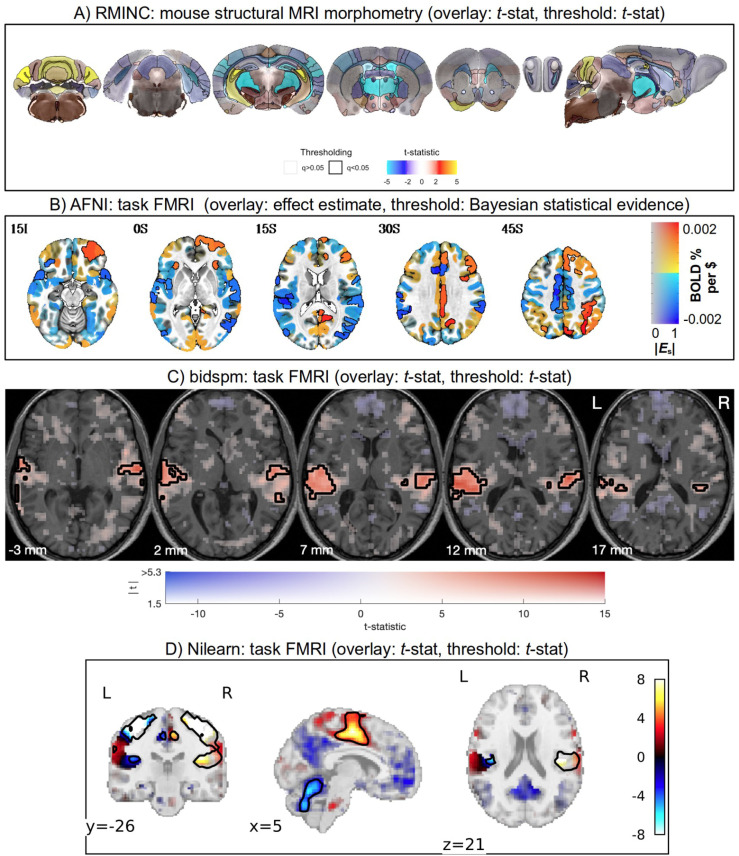
Example images of transparent thresholding from various software implementations (and see Figs. 5 and 6 for more examples). Descriptions of the data and software usage are provided in the Supplements.

As demonstrated below in Figs 5–7, there are now a large number of software packages that make a similar form of transparent thresholding available to researchers. These encompass implementations for volumetric, surface- and region-based studies. Several of these were added or streamlined as part of this work. This methodological accessibility is an important practical step for the neuroimaging field (and it will likely continue to grow), allowing researchers to easily adopt the same form of transparent thresholding for their image visualization.

This approach is a useful complement to data sharing (such as via Neurovault (Gorgolewski et al., 2015), OSF, or another resource), which itself is beneficial to the field, but applying transparent thresholding is distinct and important on its own. Figures have a powerful and primary role in interpreting results, and therefore they should be as informative as possible within the presented publication in order to facilitate accurate evaluation. The examples presented above have demonstrated this. Showing opaque figures-as-usual and relying on readers to download and visualize the data again separately does not accomplish this effectively.

### Reducing biases

Standard opaque thresholding, while done with well-meaning intentions, often inserts bias into subsequent analysis and meta-analysis. This practice both harms the ability to accurately assess reproducibility and acts to decrease reproducibility unnecessarily. The analyses of the NARPS data show both of these features, as directly demonstrated in Taylor et al. (2023) and the examples above, as well as indirectly noted in Botvinik-Nezer et al. (2020). As shown above, transparent thresholding showed increased reproducibility where appropriate (i.e., when results agreed but at different strengths) and showed low reproducibility where appropriate (when results disagreed or were essentially null). Thresholding is a processing choice, and these studies together strongly suggest that opaque thresholding is often detrimental to analyses and meta-analyses. Reproducibility has been a long-discussed topic in the field, and retaining context in results and figures is a clear step to help address it.

The adoption of transparent thresholding helps reduce several other biases in neuroimaging reporting, while still preserving the ability to highlight the most significant regions:

- Type II errors, which may arise from overly strict multiple comparisons adjustments (Cremers et al., 2017), are reduced because subthreshold regions (particularly those just below the cutoff) are still visible. Such regions can still be assessed in the context of other studies or prior knowledge, without simply being treated (inaccurately) as “no effect”.
- As discussed above, transparent thresholding greatly reduces incentives for *p*-hacking (Wicherts et al., 2016) to be able to report results in predetermined regions, or relatedly (and problematically) to “spin” results (Boutron and Ravaud, 2018).
- Publication bias or the “file drawer problem” exists in neuroimaging, where findings with weak or limited statistical evidence are typically not reported. The result is that many meta-analyses misrepresent assessments (Jennings and Van Horn, 2012; Ioannidis et al., 2014). Transparent thresholding offers a way that findings with weak statistical evidence can be assessed and reported more informatively. That is, having multiple related studies with visible sub-threshold responses, represents critical information to include in meta-analyses. Additionally, displaying subthreshold regions meaningfully, even when some locations have suprathreshold ones, can help reduce publication bias.
- Statistical thresholding itself is essentially a form of selective reporting, which was selected as the top factor contributing to reproducibility problems according to a recent Nature journal “reproducibility survey” of researchers (NPG, 2018). Consequently, overly stringent statistical thresholds and opaque thresholding may hinder rather than help the scientific process, as they can obscure meaningful patterns and impede the synthesis of findings across studies.

In addition to reducing those biases, the improved stability of transparent thresholding (see Ex. 3, above) greatly benefits studies of difficult to scan populations. While there has been a movement to increase group sizes, animal imaging studies (e.g., of nonhuman primates and rodents) remain small; in many cases, these have less than 10 subjects (Mandino et al., 2020), though these often have multiple sessions. In human clinical studies, group sizes also tend to be much smaller than standard research studies (Szucs and Ioannidis, 2020). The accrual of clinical participants is often limited by practical considerations of availability (e.g., rare diseases), cost (travel), complexity (health considerations and medical monitoring), and study length (drug trials). In these cases, opaque thresholding practices often result in few (or even no) regions above the statistical cut-off, potentially resulting in an inability to publish (i.e., publication bias) or an incentive to *p*-hack. Thus, the results that can be reported with typical thresholding are generally limited and tightly bound to the conditions of the sample itself, making them difficult to interpret and reproduce. (Data sharing is also typically more challenging for clinical studies.) While increased stability of results is not a substitute for having an adequately powered sample, transparent thresholding enables these smaller studies, acquired under difficult conditions, to contribute to the literature in a more meaningful way rather than adding to the “file drawer problem.” With transparent thresholding, the regions with larger uncertainty are clearly viewed as such, rather than being hidden or artificially pushed above thresholding by statistical maneuvering. At the same time, transparent thresholding helps reduce the likelihood of false negatives, which can be particularly important in clinical studies. It also parallels the trend in the clinical literature of moving away from the thresholding dichotomy, such as viewing results at multiple thresholds instead of a single arbitrary threshold (Voets, et al., 2025).

### Forward looking goals

As another important focus on figures, we note that papers are increasingly extracted and processed by algorithms. NeuroSynth (Yarkoni et al., 2011) was an early example of a tool that automatically parsed publication text and aggregated information together. Today, large language models (LLMs) and multimodal LLMs (MLLMs) are increasingly applied to databases and libraries—some even have a specific focus on medical imaging—creating summary tools from both text and figures (Bhayana, 2024; Bzdok et al., 2024). Just as retaining accurate descriptions in text improves the results of these tools, so does (or surely will) having more informative figures.

The adoption of transparent thresholding facilitates and complements meta-analyses that include more data in neuroimaging. For example, auxiliary scatterplots of statistics and effects in MVPA results have revealed interesting spatial patterns (see Fig 4 of Visconti di Oleggio Castello et al., (2017)). If whole brain results are shared and used for cross-study comparisons, it is more consistent to have the results shown across the whole brain in the first place. It would be confusing and inconsistent to see meta-analyses point out differences in regions that were hidden in the initial papers. Moreover, proposed multiverse approaches (e.g., Dafflon et al., 2022; Lefort-Besnard et al., 2024) aim to combine results from multiple processing pipelines or statistical methods for a given dataset, similar to meta-analyses. Transparent thresholding facilitates reporting such “doubly probabilistic” results across the whole brain, where one would expect different sub-tests to have meaningful evidence to be reported.

Another way to improve both cross-study meta-analyses and within-study interpretations is to include effect estimates in the results, rather than only showing statistics (Halsey et al., 2015). Many neuroimaging modalities have physical units, such as DWI, and some, like FMRI, can be scaled to have meaningful units (Chen et al., 2017; Flournoy et al., 2020). These provide separate information about the data and its modeling, including the *practical* significance of results. In most areas of science, it would be inconceivable not to include effect estimates, as they form the basis of analysis and interpretation. Leaving these measures out wastes information (Chen et al., 2022) and increases the ambiguity of results. For example, showing effect estimates provides useful evidence about whether suprathreshold locations are more likely true or false positives (as noted in Ex. 4). They also enable more meaningful comparisons in meta-analyses than ones based on statistics alone (e.g., Maumet and Nichols, 2016).

Finally, transparent thresholding directly benefits quality control (QC) efforts during both data processing and results presentation. Several examples of this were provided in Reynolds et al. (2023), where artifacts in the acquired EPI time series would likely have gone unnoticed without using transparent thresholding in the creation of seed-based correlation QC images. In both Ex. 1 and 4 here, subthreshold patterns helped distinguish when any results might likely be due to noise or artifact, reducing the risk of false positives. In these and other cases, wider results reporting and retention of context provide greater confidence and clearer interpretability of figures.

### Addressing comments, concerns and questions about transparent thresholding

While some in the neuroimaging field have been enthusiastic about using transparent thresholding to present more informative and less ambiguous results, others have been skeptical or raised critiques. Here we summarize some of the latter and address these points.

1. **Thresholding at exactly p=0.001 and FWE = 5% is rigorous, and reporting any other results will harm reproducibility with false positives.** Firstly, the proposed transparent thresholding still highlights the exact same regions above a given threshold (with full opacity and outlining). Secondly, threshold values themselves are typically set by convention, with various round numbers argued for at various points in history. Fisher initially wrote about *p* = 0.05 as useful, primarily because it corresponds to a round, two-tailed test value Z ≈ 2, but he also used other values such as *p* = 0.01 (Fisher, 1925). Many of his contemporary statisticians viewed such threshold choices as arbitrary and obfuscating (see Kennedy-Shaffer, 2019). More recently, one group of statisticians pushed to lower the canonical *p*-threshold to a still different value 0.005 (Benjamin et al., 2018), while another proposed rejecting *p*-value thresholds altogether (Amrhein & McShane, 2019). Even for clinical FMRI, there is no standard thresholding practice (Voets et al. 2025). This snapshot of a hundred-year-old debate alone shows there is no single, canonical threshold between “significance” and “insignificance,” and many modern statisticians view *p*-values as an unreliable focus (Halsey et al., 2015). Entirely hiding a cluster that has FWE= 5.01% is arbitrary and unscientific—such a practice itself actually harms reproducibility in the long term.
2. **A given threshold may be arbitrary, but if everyone uses the same value, then results will still be on equal footing for comparisons.** FMRI and many other kinds of neuroimaging data are noisy, with noise profiles changing across the field of view and with the scanner used. The underlying biological responses are continuous with varying magnitudes. In practice, no threshold will be consistent across studies, e.g. due to differing sample size and power, or noise characteristics and variability, or evolving acquisition strategies. Instead, applying a strict threshold for a continuous response variable also greatly increases sensitivity to non-physiological differences across studies, such as scanner type, field strength, number of subjects (see Ex. 3 above), voxel size, trial number/length, field inhomogeneities, modeling methods, etc. Opaque thresholding will strongly bias comparisons and meta-analyses towards irreproducibility and non-replicability (see the NARPS data discussion, above).
3. **Science is about “storytelling” and transparent thresholding complicates the story of results by showing more things.** Few stories of note have only main actors and no supporting cast and context—*Romeo and Juliet* (Shakespeare, 1597) would be a poor play if it contained no other characters. Perhaps fully *un*thresholded results are overly complicated to interpret for most, but transparently thresholded ones primarily add just near-significant regions and larger context. If there are not many near-threshold results, then the story stays terse. Alternatively, if there *are* many near-threshold results, then that is *part* of the story. In either scenario the reader learns from seeing the additional context. It will both facilitate storytelling and clarify a narrative, such as by suggesting which network a cluster belongs to. In Ex. 1, the additional context from transparent thresholding presents compelling evidence to challenge the narrative of strong laterality that opaque thresholding would have produced. As Einstein (probably) noted about science, “Everything should be made as simple as possible, but not simpler” (Shapiro, 2006). Storytelling should not be used as an excuse to sacrifice meaningful information.
4. **We applied transparent thresholding, and now see too many regions to discuss—the paper will be too long.** It does not seem necessary to have a detailed discussion about every single region with visible, subthreshold results. In many papers, researchers do not even write about each suprathreshold region. It would be logical to highlight any regions of particular interest, as determined from either prior research or background knowledge, and then the rest can remain as observable context and/or for potential relevance to future studies. Transparent thresholding also allows for further discussion of regions that show *no* visible response with transparent thresholding, especially if activity had been hypothesized there. In total, this should not make discussions more burdensome but instead more informative and clearer. It should also facilitate connecting the results to those of existing literature, functional networks and prior domain knowledge, by providing more globally informative results. (Additionally, if authors are concerned that a figure will not get published with transparent thresholding because there is a mess of blobs and artifacts near-threshold, this is a problem with their data and not something to be swept under the rug with opaque thresholding. Hiding issues in data to facilitate publication is generally considered poor scientific practice.)
5. **We checked out the results at multiple thresholds within our group, so we feel confident about publishing with standard thresholding.** Readers engage with scientific articles critically, a procedure that is facilitated by seeing more of the underlying evidence for themselves as they read. If transparent thresholding reveals no near-threshold results, then that simply reinforces the authors’ interpretation—nothing is lost. If transparency reveals some additional locations of interest, then readers will be aware even if it does not figure strongly into the authors’ interpretation. In fact, transparent thresholding should be viewed as a *helpful* tool to convince readers. For example, if the authors have used multiple thresholds and confirmed for themselves that their suprathreshold region is not just an extension of a near-threshold blob from the CSF, then they only strengthen their argument by including this information in their figures. In science, it benefits both the authors and readers to present the full picture. Moreover, as shown in Ex. 3, transparent thresholding will reduce the hypersensitivity of near-threshold results to arbitrary parameters (like number of subjects) and the incentives for *p*-hacking around varied thresholds.
6. **We will upload the unthresholded results to a public repository, so we will publish opaque images and people can explore the data themselves later.** Making full results public is great^5^ and certainly enables meta-analyses, but separating the more complete results from the paper greatly diminishes the ability for critical understanding by the reader. It also places a burden of time and effort that not every reader will go through.^6^ A study should provide strong evidence for its interpretations, and transparent thresholding does a better job of this than opaque thresholding. Two well-known aphorisms apply:
  - *A picture is worth a thousand words.* Figures are likely the most important messengers in a scientific study. We provided multiple examples here where there have been critical misinterpretations of written results, simply because figures were either lacking important information or were themselves missing. A summary figure can end up in talks and general discourse in ways that separate it from the complete story of the actual data in the repository and can even distort the intended message of the authors.
  - *First impressions are the most important.* Reanalysis of public data might lead to a different interpretation from an initial study, but there will be a long lag before that update can enter the scientific conversation. Consider the widely cited NARPS study, which created a strong impression of poor FMRI reproducibility that is still echoed today. The follow-up analysis of the public data by Taylor et al. (2023) showing strong evidence for a different message was published three years later, in a separate journal, and with much less impact.
7. **I’m a clinician. I need to know definite regions.** You are the expert for your work, so *you* should be the decision maker from a reasonable set of evidence. Transparent thresholding presents meaningful results for you to interpret and from which to make informed judgments. In contrast, starting with opaque thresholding presents a predetermined decision based on an arbitrary cut-off and removes potentially useful information from your consideration—in short, it puts the scalpel in the hands of an academic. It is more scientific (and likely better for clinical outcomes) to share contextualized evidence for clinicians to interpret for their purposes. Some diagnostic specialties even use fully *un*thresholded maps regularly, but when greater digestibility is required, transparent thresholding helps reduce the risk of false negatives and misinterpretation. Consider the divergent laterality findings in Ex. 1. In special cases that a binarized image is needed (e.g., in surgery), then that can still be derived from a transparently thresholded one and likely with more confidence about the localization. Voets et al. (2025) discuss further issues for clinical applications.
8. **I write for a non-technical audience, and I need to show a direct story for those outside the field.** Even for non-neuroimaging readers, oversimplifying the results is problematic. Experience with the dead salmon (Ex. 4, above) and other studies has shown that. That manuscript made a clear and valid point, but sharing only the one opaquely thresholded image has resulted in repeated misinterpretations of their core message. With transparent thresholding and the other features discussed above, we posit that the dead salmon study would have been less easy to misinterpret. While its coverage in non-technical media might not have become as expansive, those it did reach would have gained a better understanding of science and the core issues. (And it would still have received wide attention within the field, because it is a great demonstration of a serious issue.) Scientific understanding should be as accurate as possible, for both those in the field and those outside of it.
9. **Reviewers have criticized showing subthreshold results, complained about seeing more complicated spatial patterns, and do not like the lines around regions—this makes me hesitant to try to publish with this approach.** While many researchers have successfully adopted transparent thresholding in the publications (see a partial list in Table S1), it does take time for new ideas to become accepted and commonplace. We hope that articles like this, which show transparent thresholding’s many benefits and demonstrate the significant problems with opaque thresholding, will help this way of displaying results become more commonplace. If spatial patterns are complicated because many results are slightly subthreshold, then it is likely even more important to use transparency, to reduce the hypersensitivity to non-physiological features and chance of misinterpretation, as shown above in Ex. 3. While transparent thresholding is not currently normative, it is plausible that manuscripts with opaque thresholding should or will eventually themselves be critiqued over what is *not* shown to readers. We hope that the examples and points raised in this paper, as well as those in Allen et al. (2012), Chen et al. (2022), Taylor et al. (2023), and Sundermann et al. (2024), can provide convincing rationales to the reviewers for this approach.
10. **It is too difficult to implement this visualization.** Transparent thresholding in data visualization is now available in a wide number of publicly available neuroimaging software packages (as well as in separately programmed implementations): in the original Trends-Matlab toolbox (https://trendscenter.org/x/datavis; Allen et al., 2012) and the related GIFT (http://trendscenter.org/software/gift); in AFNI (Cox, 1996) and preliminarily in the surface-based visualization of SUMA (Saad et al., 2004; Saad and Reynolds, 2012); in BrainVoyager (Goebel, 2012); in FSL’s FSLeyes (McCarthy, 2024; Smith et al., 2004); in NiiVue (Hanayik et al., 2023); in RMINC (Lerch et al., 2017) and the related MRIcrotome (https://github.com/Mouse-Imaging-Centre/MRIcrotome); in CIVET (Ad-Dab’bagh et al., 2006) and the related minc-toolkit-v2 (https://github.com/BIC-MNI/minc-toolkit-v2); in Nilearn (Nilearn contributors, 2025); and in bidspm (https://github.com/cpp-lln-lab/bidspm). Representative images are shown in Figs. 5–7, which encompass volumetric, surface-based and ROI-based cases. See Table S1 in the Supplements for further examples.^7^ We hope that increased use of transparent thresholding will see its implementations spread further.

## CONCLUSION

Choosing how to threshold results is an important processing choice in FMRI and more widely across neuroimaging, including in many clinical applications. Transparent thresholding highlights the same strong regions as standard opaque thresholding but also retains brainwide context that is important for both authors and readers to see. This approach enhances understanding and helps to avoid misinterpretation in figures, which are key to presenting study results. It also provides a better framework for accurately comparing datasets and evaluating reproducibility. In contrast, opaque thresholding introduces strong biases and hypersensitivity to non-physiological features, harming within-study evaluation and cross-study reproducibility. Transparent thresholding is straightforward and easily implementable. In fact, it has already been included in a large number of packages and available scripts, providing an accessible and common way for neuroimagers display data. We hope that researchers in the field will move toward adopting this strategy when presenting results.

## Supplementary Material

Supplement 1

## Footnotes

1 It also shows the importance of including at least some images of the underlying study datasets themselves in comparisons report. No figures of individual teams’ results were shown in the NARPS paper or its supplements, though the datasets were made publicly available.

2 We note that any formal meta-analysis here would be limited, since teams were only required to upload statistics data. This follows the unfortunately common practice that effect estimate information is rarely reported, even though it has many uses (Chen et al., 2017). With more detailed effect size data, a more accurate meta-analysis could have been conducted, such as with hierarchical modeling.

3 This study pre-dates the publication of the transparent thresholding idea by Allen et al. (2012).

4 The dataset from the original dead salmon study has been lost “upstream,” but this presents the opportunity to verify that the original findings replicate in another dead fish.

5 When possible—not all datasets can be shared, particularly in clinical studies.

6 An earlier version of this draft purposely omitted images of the original Anscombe Quartet for readers to look up online, as a simplified example of the burden caused by separating relevant figures from the main text. However, even this simple search (no downloads or software needed) was deemed too frustrating by coauthors, so the images were included here.

7 Interestingly, the majority of these studies that used transparent thresholding also presented effect estimates as overlays, providing further useful information as recommended here (see Ex. 4).
