# Supplementary material for: Go Figure: Transparency in neuroscience images preserves context and clarifies interpretation
Source: ArXiv. 2025 Apr 10:arXiv:2504.07824v1. Preprint. [Version 1] (PMC12036441)
Supplement: Supplement 1 [file NIHPP2504.07824v1-supplement-1.pdf]

SUPPLEMENTS

Background: showing more results and transparent thresholding

Suggestions for displaying subthreshold results in neuroimaging go back at least two decades. Jernigan et al. (2003) suggested colored bands of statistics, and Luo and Nichols (2003) created explorable montages that included unthresholded data. Allen et al. (2012) placed outlines around suprathreshold regions and then displayed the remaining results with transparency increasing as the magnitudes shrunk ("transparent thresholding"). Bowring et al. (2019) suggested colored bands of effect confidence intervals. Pernet and Madan (2020) proposed several ideas, including displaying unthresholded data. Sundermann et al. (2024) used both transparency and different color ranges.

While not yet the standard format for image creation, transparent thresholding has been usefully applied in many neuroscience studies. With the goal of helping researchers see how transparent thresholding has been used in practice, we list a subset of peer reviewed publications that have used transparent thresholding in Table S1. Most were found using GoogleScholar to reference Allen et al. (2012) and/or Taylor et al. (2023). Studies cover a wide range of designs for FMRI, structural MRI and even non-MRI modalities, as well as studying both human and nonhuman subjects. We note that even for FMRI, several of these studies all displayed effect estimates (instead of simply statistics) as overlay maps, which was also recommended in the main text for more informative results reporting.

Reference	Comment
Sadagopan et al. (2015)	Task-based FMRI of marmosets at 7T, overlaying effect estimates
Russ and Leopold (2015)	Video watching FMRI of macaques at 4.7T, individual subject maps
Pollmann et al. (2016)	Task-based of reward, voxelwise maps, overlaying effect estimates
Axelrod (2016)	Task-based FMRI, voxelwise contrast maps, overlaying effect estimates
Guevara et al. (2017)	Resting state FMRI of newborn rats, connectivity with HbR contrast
Cohen et al. (2018)	Structural MRI, individual-to-group VBM, overlaying effect estimates
Arend et al. (2018)	Structural MRI, group-based VBM, overlaying effect estimates
van Lieshout et al. (2018)	Task-based FMRI, voxelwise contrast maps, overlaying effect estimates
Richter et al. (2018)	Task-based FMRI, voxelwise contrast maps, overlaying effect estimates
Ellingson et al. (2018)	Task-based FMRI, voxelwise contrast maps, overlaying effect estimates
Keidel et al. (2018)	Naturalistic FMRI, voxelwise contrast maps, overlaying effect estimates
Dojat et al. (2018)	Structural MRI, group-based VBM, overlaying effect estimate contrast
Ziontz et al. (2019)	^{18}F-AV-1451 PET, SUVR-based modeling, overlaying GLM coefficients
Martinez-Saito et al. (2019)	Task-based FMRI, voxelwise contrast maps
Ai et al. (2019)	Structural MRI, multiple regression analyses, overlaying effect estimates
Chaze et al. (2019)	MR elastography, stiffness maps
Polsek et al. (2020)	Structural MRI of rodents at 7T
Handwerker et al. (2020)	Resting state FMRI and theta-burst TMS, correlation changes
Hofmans et al. (2020)	Task-based FMRI, voxelwise coefficients, overlaying effect estimates
Dellert et al. (2021)	Simultaneous EEG-FMRI, PPMs, overlaying effect estimates
Brolsma et al. (2021)	Task-based FMRI, voxelwise GLMs, overlaying effect estimates

van den Bosch et al. (2022)	Task-based pharmaco-FMRI, overlaying effect estimates
Böttinger et al. (2022)	Task-based FMRI, voxelwise contrast maps, overlaying effect estimates
van den Bosch et al. (2023)	Task-based pharmaco-FMRI, overlaying effect estimates
Boulakis et al. (2023)	Resting state FMRI with experience sampling
Orwig et al. (2023)	Resting state FMRI with GLMs and network analysis
Bishay et al. (2024)	FDG-PET, assoc. of chemoradiation and change in glucose metabolism
Strigo et al. (2024)	Task-based FMRI, voxelwise association maps
Reddy, Zvolanek et al. (2024)	Task-based ME-FMRI, voxelwise contrasts,
Florito et al. (2024)	Meta-analysis of FMRI, overlaying effect estimates (mean Hedges' g)
Kinsey et al. (2024)	Task-based FMRI, voxelwise contrast maps, overlaying effect estimates
Coursey et al. (2024)	FDG-PET and FMRI, various measures, surface-based analyses
Reddy, Clements, et al. (2024)	Task-based ME-FMRI, voxelwise contrasts, overlaying effect estimates
Beynel, et al. (2024)	Task-based FMRI, voxelwise contrasts, overlaying effect estimates
Avery, et al. (2025)	Task-based FMRI, voxelwise contrasts
Mantas et al. (2025)	Structural MRI of mice at 9.4T, TBM assoc., overlaying effect estimates
Freund et al. (2025)	Task-based FMRI, test-retest reliability, ROI-based surface analysis
Aloi et al. (2025)	Resting state FMRI, group differences in functional correlation

Table S1. *A non-exhaustive list of studies using transparent thresholding in figures. The majority of these studies also display effect estimates as overlays, rather than just statistics, which provides useful information (as discussed in the main text).*
VBM = voxel-based morphometry. PET = positron emission tomography. SUVR = standardized uptake value ratios. GLM = general linear model. TMS = transcranial magnetic stimulation. PPM = posterior probability maps. FDG = fluorodeoxyglucose. ME = multi-echo. TBM = tensor-based morphometry

FMRI data and processing

Related data for the examples are freely available via OSF (https://osf.io/n4a37). Processing and visualization scripts are freely available via GitHub, with links provided for each case below.

The task-based FMRI data for Ex. 1 have been previously acquired and described; see Smith et al. (2020) and Chen et al. (2021) for details. Briefly, 42 healthy youth and adults were scanned while performing a modified Eriksen Flanker task with two stimulus classes, congruent and incongruent. Data were acquired in two separate sessions with a total of 8 runs, containing 432 trials for each of the two conditions (of which only trials with correct responses were used in the analysis). Echo-planar images (EPIs) had: flip angle=60°, TE=25 ms, TR=2000 ms, 170 volumes per run, voxel=2.5x2.5x3.0 mm^3. Accompanying structural anatomical was acquired at 1mm isotropic resolution using T1-weighted a standard magnetization-prepared rapid acquisition gradient echo (MPRAGE) sequence. The data were processed using AFNI (Cox, 1996) version 20.3.00. Variations of the processed data (shown in Fig. 1B) were created as follows: 3dcalc was used to negate and zero the left hemisphere (Panels 2-3, respectively); a combination of 3dClustSim and 3dcalc were used to create Panel 4.

The task-based FMRI data for Ex. 2 and 3 were previously acquired as part of the NARPS project (Botvinik-Nezer et al., 2020); see that paper for acquisition details. Briefly, two groups of 54 subjects each performed variations of a mixed gambling paradigm task while being scanned, responding to potential gains and losses at various levels; there were four runs per subject. The EPI data were acquired with: flip angle=68°, TE=30ms, TR=1000ms, multiband=4, parallel factor (iPAT)=2, voxel size=2.0x2.0x2.4 mm^3, 453 volumes per run. Each subject's accompanying structural T1-weighted MPRAGE dataset had 1 mm isotropic resolution.

The group data used in Ex. 2 are publicly available through NeuroVault (Gorgolewski et al. 2015). Participating teams uploaded either *t*- or *Z*-statistic maps, which were unthresholded. The original NARPS data collection had 54 subjects in each of the two groups. Taylor et al. (2023) reported having 47 and 52 subjects per group after processing and QC, and we estimated that most teams' results would also have approximately 50 subjects remaining per group. Thus, the resulting degrees of freedom (DFs) would be enough to apply the same threshold value to either statistical dataset and consider it approximately equivalent (Fig. 2).

The FMRI processing for data used in Ex. 3 is described in Taylor et al. (2023), with accompanying GitHub repository of scripts (https://github.com/afni/apaper_highlight_narps). Standard processing included FreeSurfer (Fischl and Dale, 2000) version 7.1.1 and AFNI (Cox, 1996) version 22.2.12, particularly using afni_proc.py for full FMRI processing and quality control (Reynolds et al., 2024; Taylor et al., 2024).

In Figs. 2 and 3, similarity matrices are estimated to summarize properties across datasets in the cases where strict thresholding is applied or not. When opaque thresholding is applied, the results are binarized maps of suprathreshold regions, and therefore the Dice coefficient is appropriate for quantifying overlaps. In more formal analyses, separate Dice calculations would be made for sets of positive and negative regions (e.g., see Taylor et al., 2023), where reduced and similar values were observed, respectively; for simplicity here, all regions were put into a Dice comparison. For continuous value maps that exist when thresholding is not applied, Pearson correlation is appropriate to quantify the patterns in the data. Both Dice and Pearson similarities are straightforward, clear and widely used for the respective kinds of data. Conveniently, they also both have a maximum of 1 and minimum magnitude of 0 (with Pearson offering separate information of anticorrelation). We note that there may be other ways to quantify similarity. For example, for the continuous values one might choose to weight the correlations by the magnitude of the statistics themselves, thus providing greater influence to the high-statistic values in a fashion that more closely mimics the "highlighting" visualization of transparent thresholding; one could even use the alpha values that are calculated in the transparent thresholding visualization as weights. Either of these might be reasonable as high-statistic regions are often of the greatest interest. Separately, Gorgolewski et al. (2012) proposed an iterative approach of thresholding and Dice estimation to find a threshold that maximizes similarity.

In Ex. 4, previously acquired data by Bennett et al. (2009) of the "dead salmon" were no longer available (a query was made to the study authors, but they could not find the data). Therefore,

the sole image from the original publication was used (in Fig. 4A and 4B-left), and an altered version showing what the "after correction" results would look like with strict thresholding was derived from that (Fig. 4B-right).

To present a more complete discussion of these results, a new "dead salmon" dataset was acquired with a task-based FMRI paradigm using a General Electric MR-750 3T MRI scanner. These data were collected in accordance with all local regulations and facility stipulations. Two runs of EPI data from a single *ex vivo* salmon (in a container with fluorinert to remove air pockets and gauze to minimize motion from scanner vibrations) were acquired with: flip angle=75°, TE=25 ms, TR=2000 ms, 170 volumes per run, voxel=2.0x2.0x2.5 mm^3. The accompanying structural T1-weighted MPRAGE dataset had 0.625x0.625x1.0 mm^3 resolution. The presented task stimulus was a standard flashing checkerboard with a 10-second duration, and there were 20 trials total across the two runs. Standard processing included AFNI version 24.1.05, particularly using afni_proc.py for full FMRI processing and quality control (Reynolds et al., 2024; Taylor et al., 2024). The full processing and visualization scripts are available (https://github.com/afni/apaper_gofigure_salmon), and demo downloads of both the raw and fully processed (including APQC HTML reports) are available at the project's OSF page (https://osf.io/n4a37).

Brain images in the main text: displaying overlays and applying transparent thresholding

The brain images displayed in Figs. 1-4 of the main text were created using AFNI (Cox, 1996), specifically using the toolbox's @chauffeur_afni command line program. This program facilitates systematic image and montage generation, by making many AFNI GUI and environment features scriptable, which is useful for figure generation and code sharing. The variously thresholded and faded colorbars were edited using AFNI's colorbar_tool.py program.

When displaying overlay data, AFNI allows for separating the dataset that is displayed (the "overlay" dataset, whose values are represented by colorbar mapping) from that which is thresholded (the "threshold" dataset, whose values are used to determine whether a voxel's overlay value will be displayed, or with what opacity in the case of transparent thresholding). For example, with opaque thresholding, a voxel's overlay dataset value would only be displayed if the corresponding threshold dataset value were suprathreshold; otherwise it would not be displayed. This partitioning of properties allows for the useful and common case of displaying effect estimates as overlay coloration while thresholding based on statistical information.

While it is still unfortunately common practice in the neuroimaging field to ignore effect estimate values and use statistics for both the overlay coloration and threshold, the benefits of visualizing the distinct effect information in results has been shown (Chen et al., 2017). The latter has been used here in Figs. 3-4, where per-voxel scaling has been applied to transform the unitless EPI data into meaningful BOLD percent signal change, which was then displayed as the overlay (while the accompanying statistic was used for thresholding). Only statistical information is available for each team's results in the public NARPS data, so Fig. 2 could only use this for both the overlay and threshold data. In Fig. 1, only the statistic is also used for both to not distract

unaccustomed readers to the main point of the example. In the future, we hope that displaying effect estimates becomes more common practice, providing more information to readers and facilitating meta-analyses.

To perform transparent thresholding, AFNI implements a similar formulation as Allen et al. (2012). The transparency of an overlay voxel is calculated from the chosen "threshold" dataset, defining what is commonly termed the "alpha opacity", which is a continuous value between 0 and 1, inclusively. For a threshold dataset voxel of magnitude M and a selected threshold T, we have the following basic implementation with a quadratic decrease of alpha by default: if M>=T, then alpha = 1; else, alpha = (M/T)**2; users can also select a linear decrease in opacity, alpha=M/T, within the AFNI GUI, environment, or @chauffeur_afni program. The alpha value is then applied as a simple mixing fraction of the underlay and overlay RGB color vectors: RGB_final = (1-alpha)*RGB_ulay + alpha*RGB_olay. Thus, if alpha=1, the overlay is opaque (no underlay coloration is seen), while for intermediate values the overlay color fades into the background. As opposed to simply applying no thresholding to the overlay, this helps highlight regions of highest statistical significance (or of whatever dataset is applied as the "threshold" volume) while still allowing near-threshold results to be appreciated.

As a further highlighting measure, users can also place an outline around the suprathreshold regions, again following (Allen et al., 2012). It is default to use a black line, but AFNI allows a wide variety of other colors to be selected, as well, to maximize suitability across varied applications. For example, white outlines were used in Figs. 3 and 4.

Software implementations and visualization

Figs. 5 and 6 in the main text provides example illustrations of transparent thresholding across various software packages and implementations. We briefly describe the data and any relevant comments here.

The colorbars in these (and other) figures typically represent the use of transparent thresholding by fading to a background grayscale color (light gray or black). The fading can be either a linear or quadratic function of subthreshold value, as chosen by the researcher and/or software package implementation. When the overlay and thresholding datasets are the same, the fading is applied along the colorbar gradient itself. When the overlay and thresholding datasets differ (e.g., using an effect estimate for the former and a statistic for the latter), then the fading is orthogonal to the color gradient. Most images also include a black or white boundary around the suprathreshold regions; some software have yet to implement this fully, but are in process on doing so.

SUMA. This example (Fig. 5) uses a preliminary implementation of transparent thresholding in SUMA's surface mapping visualization (Saad et al., 2004; Saad and Reynolds, 2012). The image displays single subject results from a task-based FMRI study on an inflated surface representation, where the underlay grayscale shows the sulcal and gyral patterns. The data and processing scripts are part of the publicly available AFNI Bootcamp teaching material

(https://afni.nimh.nih.gov/pub/dist/doc/htmldoc/background_install/bootcamp_stuff.html; https://www.youtube.com/c/afnibootcamp). Specifically, the script *s03.ap.surface* was used to run afni_proc.py (Reynolds et al., 2024) to process the data and project it onto a FreeSurfer-estimated (Fischl and Dale, 2000) surface mesh that has been standardized using AFNI's @*SUMA_Make_Spec_FS*. The task FMRI data consisted of three runs of 150 time points each, during which a block design stimulus paradigm of "visual reliable" and "audio reliable" tasks were presented. The figure shows the full F-stat results (degrees of freedom: 2, 412) of the modeling, transparently thresholded at F=25. As with AFNI's implementation, the subthreshold fading of opacity is quadratic by default (as shown in Fig. 5), but can also be changed to linear.

NiiVue. This example (Fig. 5) uses the open source NiiVue (Hanayik et al. 2023) library to display single subject modeling results from a resting state study acquired with multi-echo (ME) FMRI data. NiiVue visualization is supported in all major web browsers, and enables application developers to create easily accessible web pages to publish data using these visualization techniques. This dataset is available as part of AFNI's ME-FMRI demo (downloadable via the command @Install_APMULTI_Demo1_rest; Taylor et al., 2022), and its acquisition and applications are described more in Gilmore et al. (2019) and in Gotts et al. (2020). The script *do_25_ap_me_br.tcsh* was used to run afni_proc.py (Reynolds et al., 2024) to process the data, including optimal combination of echos (Posse et al., 1999) as implemented within AFNI. By default, NiiVue uses an HTML canvas element to display volumetric data in a multiplanar layout with an optional 3D volume rendering tile. Each imaging plane can be navigated independently and volume rendering supports an interactive clip plane. The image displays Pearson correlation as for both overlay coloration and thresholding, with the transparent thresholding applied at $|r|$=0.3; NiiVue uses quadratic alpha fading, as in AFNI's default (described above). This panel is part of the afni_proc.py quality control (APQC) HTML (Taylor et al., 2024), which includes such toggleable Niivue instances for within-browser exploration (see an interactive online demo at https://afni.github.io/qc-demo-repo/). Additionally NiiVue's mesh visualization also contains transparent thresholding functionality for multilayered surface displays.

AFNI. This example (Fig. 5) uses AFNI (Cox, 1996) to display modeling results from a single macaque in task-based FMRI study. The data and scripts are available as part of a full processing demo (downloadable via the command @Install_MACAQUE_DEMO), which are described along with the acquisition paradigm in (Jung et al., 2021). The script *do_20_ap.tcsh* was used to run afni_proc.py (Reynolds et al., 2024) to process the data. The task data consisted of four runs of 112 time points each, during which a block design task of image presentation (faces, objects, scrambled faces and scrambled objects) were presented; MION (monocrystalline iron oxide nanoparticle; Vanduffel et al., 2001) was also applied as a contrast agent, and this was accounted for during processing. The displayed image shows the "(intact images) - (scrambled images)" contrast as the overlay in units of BOLD % signal change and the associated t-statistic as the thresholding volume. Transparent thresholding is applied at $|t|$ =3.3, corresponding to p=0.001. This montage is part of afni_proc.py's APQC HTML (Taylor et al., 2024), to facilitate systematic checks of data. Transparent thresholding is particularly helpful to see potential subthreshold artifacts that may be part of the data (Reynolds et al., 2023).

An example of using AFNI to visualize transparent thresholding in an ROI-based analysis is also provided (Fig. 7). The image shows results of Bayesian multilevel modeling with AFNI's RBA program (Chen et al., 2019), for the task-based FMRI analysis of Hypothesis 2 of the NARPS dataset. The effect estimate value in units of BOLD percent signal change per dollar (as the paradigm included a gambling-related task stimulus) is shown within each ROI of the utilized Glasser atlas (Glasser et al., 2016). The values used for thresholding are the statistical evidence E_s from the Bayesian modeling, essentially the posterior probability P^+, scaled to be in range [-1, 1]. Thresholding was applied at $|E_s| > 0.95$, with suprathreshold regions outlined in black and transparency fading at a quadratic rate. This image was created using a new program in AFNI called chauffeur_map_rois, which wraps around @chauffeur_afni.

FSLeyes. This example (Fig. 5) uses FSLeyes (McCarthy, 2024) to display single-subject modeling results from a task FMRI study, acquired as part of the publicly available FSL Course data set (https://open.win.ox.ac.uk/pages/fslcourse/website/). During the experiment, words were presented at different frequencies. Sentences were presented one word at a time, at frequencies ranging from 50 words per minute (wpm) to 1250 wpm, and the participant simply had to read the words as they were presented. The displayed image shows the results from a *F*-test comparing all pairs of frequencies, which was therefore sensitive to brain regions that responded differently to different word frequencies. Cluster-based thresholding was used to identify significant regions, with a cluster-forming threshold of Z=3.1 and cluster significance threshold of p=0.05. The example shows axial slices from the corresponding Z-statistic image, with the black outline highlighting significant clusters. Voxels with a Z-value at or above 3.1 are fully opaque, whereas the opacity of voxels with Z-value below 3.1 is linearly modulated by the Z value.

This visualisation can be achieved in FSLeyes for any statistic from a FSL FEAT analysis (Smith et al., 2004) by:

1. loading the un-thresholded and thresholded statistic images (e.g. *stats/zfstat1.nii.gz* and *thresh_zfstat1.nii.gz*), along with a suitable background image (e.g. *example_func.nii.gz*).
2. For the thresholded image, setting the *Overlay type* to *Mask*, and enabling the *Show outline only* option.
3. For the un-thresholded image, disabling the *Link display/clipping ranges*, and enabling the *Modulate alpha by intensity* option.
4. For the un-thresholded image, adjusting the positive and negative colour maps, and display and modulate range, as desired.

FSLeyes also allows the transparency of one image to be modulated by that of another image via the *Modulate by* option. This option allows one to, for instance, display contrast of parameter estimate (COPE) values and have their transparency modulated by the corresponding Z-statistics.

Trends-Matlab & GIFT. This example (Fig. 6) uses Matlab-based scripts (see https://trendscenter.org/x/datavis; Allen et al., 2012) to visualize task-based FMRI group results from a study by Kinsey et al. (2024). Comparisons between explicitly nonlinear (ENL) subject-

level temporal (TEMP) and posterior default mode (pDM) intrinsic connectivity network (ICN) estimates derived from healthy controls (HC) and individuals with schizophrenia (SZ). Results are plotted according to a dual-coded colormap with transparency reflecting two-sided independent-samples *t*-statistic magnitudes (n = 508; df = 506), and contours indicate false discovery rate-corrected statistical significance ($q < 0.05$), as well as the outer edge of the masked EPI data. Warmer hues indicate HC > SZ, and cooler hues indicate SZ > HC. Results are overlaid on the ch2bet template with x, y and z coordinates listed relative to the origin in Montreal Neurological Institute 152 space. This thresholding approach is also implemented in the group ICA of fMRI toolbox (GIFT; http://trendscenter.org/software/gift).

BrainVoyager. The possibility to use transparent thresholding is available as a standard feature in BrainVoyager (Goebel, 2012). Contours of the thresholded map can be created using the 'Convert Map Clusters to VOI(s)' option. The upcoming version 24.0 release of BrainVoyager will perform the contouring as default when turning on transparent map thresholding. The displayed example (Fig. 6) uses the NeWBI4fMRI tutorial 1 dataset (see https://www.newbi4fmri.com/tutorial-1-data), which is a single participant's data. The task-based FMRI stimulation consisted of faces, hands, bodies, scrambled images, and fixation baseline conditions. The transparent thresholding example visual is created using 'General Linear Model: Single Study' with default parameters as implemented in BrainVoyager version 24, and it displays all stimulation conditions versus the fixation baseline contrast. The transparent coloring overlaid on the greyscale anatomical image reflects the statistical values below the chosen threshold q(FDR) < 0.05. The opaque black lines represent the statistical values above the q(FDR) < 0.05 threshold only at the borders of the spatial clusters.

CIVET & minc-toolkit-v2. The image (Fig. 6) was created with colour_object/ray_trace from the minc-toolkit-v2 (https://github.com/BIC-MNI/minc-toolkit-v2). Vertex-wise cortical thickness was estimated using CIVET (Ad-Dab'bagh et al., 2006), comparing the effect of first episode psychosis group vs a control group, with age and sex covariates. (The datasets come from unpublished work from the Prevention and Early Intervention for Psychosis (PEPP) clinic in Montreal, Canada.) Both the colormap and threshold volume are the *t*-statistic from the modeling. An arbitrary threshold of $|t| = 2$ has been applied, below which transparent thresholding occurs with a linear fade. No boundary contour has been placed around the supratheshold regions, but there are plans to add this functionality.

RMINC & MRIcrotome. The image in Fig. 6 was created in the R-programming language, by using RMINC (Lerch et al., 2017) and the related MRIcrotome (https://github.com/Mouse-Imaging-Centre/MRIcrotome). The structural MRI data from Wistar rats were analyzed using deformation-based morphometry (DBM), as part of a study of the longitudinal effects of morphine self-administration (n = 33) versus a control group (n = 36), from postnatal day 60 (P60) to 81 (P81). The maps are the result of a 2x3 linear mixed model, for the interaction effect (Group [Morphine vs Control] x Time [T1,T2,T3]). The overlaid colormap describes the direction of the *t*-statistics: cool colors denote negative values compared to the control group, and warmer colors denote positive values. The transparent threshold is set at $|t| = 2.5$ (black line), which is the adjusted value for FDR = 5%, and fades linearly with decreasing statistic. Locations

with even more significant statistic values are additionally highlighted with a yellow boundary (|*t*| = 3.1, which is where FDR = 1%).

An ROI-based example with RMINC visualization is also provided (Fig. 7). The ROI morphometry compares two inbred mouse strains (C57BL/6J vs DBA) and shows *t*-statistics encoded by both color and alpha-based transparency. The FDR-based threshold at 5% is indicated by solid black lines.

Nilearn. The transparency thresholding feature will be available in Nilearn (Nilearn contributors, 2025) from release 0.12 (scheduled for April 2025). It can be used by passing a statistical map in Nifti format (Cox et al., 2004) to the "*transparency"* parameter in several plotting functions in Nilearn such as: *plot_glass_brain*, *plot_stat_map* and *plot_img*. Users can use the "*transparency_range"* parameter to specify the range of values between which the transparency would vary. This would make the voxels with values below this range to be fully transparent and the ones above this range to be fully opaque. Fig. 7 shows a *t*-statistical contrast map from Thirion et al. (2014) (https://neurovault.org/images/10426/) overlayed on top of an MNI ICBM152 volumetric template via the *plot_stat_map* function. The full demonstration of this feature with other data and plotting functions is available on the development version of the Nilearn documentation (https://nilearn.github.io/dev/auto_examples/01_plotting/plot_transparency.html).

bidspm. The transparent plot in Fig. 7 is created using "Slice Display" (Zandbelt, 2017) implemented in bidspm (v4.0.0 - https://github.com/cpp-lln-lab/bidspm), a Matlab/Octave toolbox to perform MRI data analyses on a BIDS dataset (Gorgolewski et al., 2016) using SPM12 (Wellcome Trust Centre for Neuroimaging London, UK). The example shown displays the results obtained with the "stats" and "results" steps processing a single subject for the "listening" condition from the "Mother of All Experiments (MoAE)" dataset (https://www.fil.ion.ucl.ac.uk/spm/download/data/MoAEpilot). The transparent plot shows the *t*-statistic contrast map overlaid onto the normalized T1w structural image (IXI549Space), the transparent thresholding is applied at |*t*| = 5.3 corresponding to a voxelwise FWE-corrected threshold of $p = 0.05$ fading with decreasing statistic, and the black line represents the contour of the suprathreshold regions. Parameters such as image plane, number of slices, result maps, and applied transparency are fully customizable by the user. A demonstration of how to implement a transparent plot from bidspm results is available from the toolbox documentation (https://bidspm.readthedocs.io/en/latest/demos/moae.html). Future releases of bidspm will implement a fully automated generation of transparent plots from the results.

ADDITIONAL REFERENCES IN SUPPLEMENTS

Ai H, Xin Y, Luo YJ, Gu R, Xu P (2019). Volume of motor area predicts motor impulsivity. Eur J Neurosci 49(11):1470-1476. doi: 10.1111/ejn.14339. Epub 2019 Jan 29. PMID: 30636081.

Aloi J, Korin TE, Murray OK, Crum KI, LeFevre K, Dzemidzic M, Hulvershorn LA (2025). Latent Profiles of Impulsivity and Emotion Regulation in Children with Externalizing Disorders are Associated with Alterations in Striatocortical Connectivity. Biological Psychiatry: Cognitive Neuroscience and Neuroimaging (in press). https://doi.org/10.1016/j.bpsc.2025.02.013

Arend I, Yuen K, Sagi N, Henik A (2018). Neuroanatomical basis of number synaesthesias: A voxel-based morphometry study. Cortex 101:172-180. doi: 10.1016/j.cortex.2018.01.020. Epub 2018 Feb 7. PMID: 29482015.

Avery JA, Carrington M, Ingeholm JE, Darcey V, Simmons WK, Hall KD, Martin A. Automatic engagement of limbic and prefrontal networks in response to food images reflects distinct information about food hedonics and inhibitory control. Commun Biol. 2025 Feb 20;8(1):270. doi: 10.1038/s42003-025-07704-w. PMID: 39979602; PMCID: PMC11842766.

Axelrod V (2016). On the domain-specificity of the visual and non-visual face-selective regions. Eur J Neurosci 44(4):2049-63. doi:10.1111/ejn.13290. Epub 2016 Jul 1. PMID: 27255921.

Beynel L, Gura H, Rezaee Z, Ekpo EC, Deng ZD, Joseph JO, Taylor P, Luber B, Lisanby SH. Lessons learned from an fMRI-guided rTMS study on performance in a numerical Stroop task. PLoS One. 2024 May 6;19(5):e0302660. doi: 10.1371/journal.pone.0302660. PMID: 38709724; PMCID: PMC11073721.

Böttinger BW, Baumeister S, Millenet S, Barker GJ, Bokde ALW, Büchel C, Quinlan EB, Desrivières S, Flor H, Grigis A, Garavan H, Gowland P, Heinz A, Ittermann B, Martinot JL, Martinot MP, Artiges E, Orfanos DP, Paus T, Poustka L, Fröhner JH, Smolka MN, Walter H, Whelan R, Schumann G, Banaschewski T, Brandeis D, Nees F; IMAGEN Consortium (2022). Orbitofrontal control of conduct problems? Evidence from healthy adolescents processing negative facial affect. Eur Child Adolesc Psychiatry 31(8):1-10. doi: 10.1007/s00787-021-01770-1.

Boulakis PA, Mortaheb S, van Calster L, Majerus S, Demertzi A (2023). Whole-Brain Deactivations Precede Uninduced Mind-Blanking Reports. J Neurosci 43(40):6807-6815. doi: 10.1523/JNEUROSCI.0696-23.2023. Epub 2023 Aug 29. PMID: 37643862; PMCID: PMC10552942.

Brolsma SCA, Vassena E, Vrijsen JN, Sescousse G, Collard RM, van Eijndhoven PF, Schene AH, Cools R (2021). Negative Learning Bias in Depression Revisited: Enhanced Neural Response to Surprising Reward Across Psychiatric Disorders. Biol Psychiatry Cogn Neurosci

Neuroimaging 6(3):280-289. doi: 10.1016/j.bpsc.2020.08.011. Epub 2020 Aug 30. PMID: 33082119

Chaze CA, McIlvain G, Smith DR, Villermaux GM, Delgorio PL, Wright HG, Rogers KJ, Miller F, Crenshaw JR, Johnson CL (2019). Altered brain tissue viscoelasticity in pediatric cerebral palsy measured by magnetic resonance elastography. Neuroimage Clin. 2019;22:101750. doi: 10.1016/j.nicl.2019.101750. Epub 2019 Mar 7. PMID: 30870734; PMCID: PMC6416970.

Chen G, Xiao Y, Taylor PA, Rajendra JK, Riggins T, Geng F, Redcay E, Cox RW (2019). Handling Multiplicity in Neuroimaging Through Bayesian Lenses with Multilevel Modeling. Neuroinformatics. 17(4):515-545. doi:10.1007/s12021-018-9409-6

Cohen ZZ, Arend I, Yuen K, Naparstek S, Gliksman Y, Veksler R, Henik A (2018). Tactile enumeration: A case study of acalculia. Brain Cogn 127:60-71. doi:10.1016/j.bandc.2018.10.001. Epub 2018 Oct 16. PMID: 30340181.

Coursey SE, Mandeville J, Reed MB, Hartung GA, Garimella A, Sari H, Lanzenberger R, Price JC, Polimeni JR, Greve DN, Hahn A, Chen JE (2024). On the analysis of functional PET (fPET)-FDG: baseline mischaracterization can introduce artifactual metabolic (de)activations. bioRxiv [Preprint]. 2024 Oct 21:2024.10.17.618550. doi: 10.1101/2024.10.17.618550. PMID: 39484579; PMCID: PMC11526866.

Cox RW, Ashburner J, Breman H, Fissell K, Haselgrove C, Holmes CJ, Lancaster JL, Rex DE, Smith SM, Woodward JB, Strother SC (2004). A (sort of) new image data format standard: NiFTI-1. Presented at the 10th Annual Meeting of the Organization for Human Brain Mapping.

Dellert T, Müller-Bardorff M, Schlossmacher I, Pitts M, Hofmann D, Bruchmann M, Straube T (2021). Dissociating the Neural Correlates of Consciousness and Task Relevance in Face Perception Using Simultaneous EEG-fMRI. J Neurosci 41(37):7864-7875. doi: 10.1523/JNEUROSCI.2799-20.2021.

Dojat M, Pizzagalli F, Hupé JM (2018). Magnetic resonance imaging does not reveal structural alterations in the brain of grapheme-color synesthetes. PLoS One 13(4):e0194422.

Ellingson LD, Stegner AJ, Schwabacher IJ, Lindheimer JB, Cook DB (2018). Catastrophizing Interferes with Cognitive Modulation of Pain in Women with Fibromyalgia. Pain Med 19(12):2408-2422. doi: 10.1093/pm/pny008. PMID: 29474665; PMCID: PMC6659027.

Fiorito AM, Blasi G, Brunelin J, Chowdury A, Diwadkar VA, Goghari VM, Gur RC, Kwon JS, Quarto T, Rolland B, Spilka MJ, Wolf DH, Yun JY, Fakra E, Sescousse G (2024). Blunted brain responses to neutral faces in healthy first-degree relatives of patients with schizophrenia: an image-based fMRI meta-analysis. Schizophrenia 10(1):38.

Freund MC, Chen R, Chen G, Braver TS. Complementary benefits of multivariate and hierarchical models for identifying individual differences in cognitive control. Imaging Neurosci (Camb). 2025 Feb 10;3:imag_a_00447. doi: 10.1162/imag_a_00447. PMID: 39957839; PMCID: PMC11823007.

Gilmore AW, Kalinowski SE, Milleville SC, Gotts SJ, Martin A (2019). Identifying task-general effects of stimulus familiarity in the parietal memory network. Neuropsychologia 124:31-43. doi: 10.1016/j.neuropsychologia.2018.12.023.

Glasser MF, Coalson TS, Robinson EC, Hacker CD, Harwell J, Yacoub E, Ugurbil K, Andersson J, Beckmann CF, Jenkinson M, Smith SM, Van Essen DC (2016). A multi-modal parcellation of human cerebral cortex. Nature 536(7615):171-178.

Gorgolewski KJ, Auer T, Calhoun VD, Craddock RC, Das S, Duff EP, Flandin G, Ghosh SS, Glatard T, Halchenko YO, Handwerker DA, Hanke M, Keator D, Li X, Michael Z, Maumet C, Nichols BN, Nichols TE, Pellman J, Poline JB, Rokem A, Schaefer G, Sochat V, Triplett W, Turner JA, Varoquaux G, Poldrack RA (2016). The brain imaging data structure, a format for organizing and describing outputs of neuroimaging experiments. Sci Data 3:160044. doi: 10.1038/sdata.2016.44.

Gotts SJ, Gilmore AW, Martin A (2020). Brain networks, dimensionality, and global signal averaging in resting-state fMRI: Hierarchical network structure results in low-dimensional spatiotemporal dynamics. Neuroimage 2020 205:116289. doi: 10.1016/j.neuroimage.2019.116289.

Guevara E, Pierre WC, Tessier C, Akakpo L, Londono I, Lesage F, Lodygensky GA (2017). Altered Functional Connectivity Following an Inflammatory White Matter Injury in the Newborn Rat: A High Spatial and Temporal Resolution Intrinsic Optical Imaging Study. Front Neurosci 11:358. doi: 10.3389/fnins.2017.00358. PMID: 28725174; PMCID: PMC5495836.

Handwerker DA, Ianni G, Gutierrez B, Roopchansingh V, Gonzalez-Castillo J, Chen G, Bandettini PA, Ungerleider LG, Pitcher D (2020). Theta-burst TMS to the posterior superior temporal sulcus decreases resting-state fMRI connectivity across the face processing network. Netw Neurosci 4(3):746-760.

Hofmans L, van den Bosch R, Määttä JI, Verkes RJ, Aarts E, Cools R. The cognitive effects of a promised bonus do not depend on dopamine synthesis capacity. Sci Rep. 2020 Oct 5;10(1):16473. doi: 10.1038/s41598-020-72329-4. PMID: 33020514; PMCID: PMC7536197.

Keidel JL, Oedekoven CSH, Tut AC, Bird CM (2018). Multiscale Integration of Contextual Information During a Naturalistic Task. Cereb Cortex 28(10):3531-3539. doi: 10.1093/cercor/bhx218. PMID: 28968727.

Kinsey S, Kazimierczak K, Camazón PA, Chen J, Adali T, Kochunov P, Adhikari BM, Ford J, van Erp TGM, Dhamala M, Calhoun VD, Iraji A (2024). Networks extracted from nonlinear fMRI connectivity exhibit unique spatial variation and enhanced sensitivity to differences between individuals with schizophrenia and controls. Nat Ment Health 2(12):1464-1475. doi: 10.1038/s44220-024-00341-y. Epub 2024 Nov 21. PMID: 39650801; PMCID: PMC11621020.

Martinez-Saito M, Konovalov R, Piradov MA, Shestakova A, Gutkin B, Klucharev V (2019). Action in auctions: neural and computational mechanisms of bidding behaviour. Eur J Neurosci 50(8):3327-3348. doi: 10.1111/ejn.14492. Epub 2019 Jul 29. PMID: 31219633; PMCID: PMC6899836.

Mantas I, Flais I, Branzell N, Ionescu TM, Kim E, Zhang X, Cash D, Hengerer B, Svenningsson P. A molecular mechanism mediating clozapine-enhanced sensorimotor gating. Neuropsychopharmacology. 2025 Feb 11. doi: 10.1038/s41386-025-02060-z. Epub ahead of print. PMID: 39934408.

Orwig W, Diez I, Bueichekú E, Kelly CA, Sepulcre J, Schacter DL (2023). Intentionality of Self-Generated Thought: Contributions of Mind Wandering to Creativity. Creat Res J 35(3):471-480. doi: 10.1080/10400419.2022.2120286.

Polsek D, Cash D, Veronese M, Ilic K, Wood TC, Milosevic M, Kalanj-Bognar S, Morrell MJ, Williams SCR, Gajovic S, Leschziner GD, Mitrecic D, Rosenzweig I (2020). The innate immune toll-like-receptor-2 modulates the depressogenic and anorexiolytic neuroinflammatory response in obstructive sleep apnoea. Sci Rep 10(1):11475. doi: 10.1038/s41598-020-68299-2. PMID: 32651433; PMCID: PMC7351955.

Pollmann S, Eštočinová J, Sommer S, Chelazzi L, Zinke W (2016). Neural structures involved in visual search guidance by reward-enhanced contextual cueing of the target location. Neuroimage 124(Pt A):887-897. doi: 10.1016/j.neuroimage.2015.09.040. Epub 2015 Sep 30. PMID: 26427645.

Reddy NA, Zvolanek KM, Moia S, Caballero-Gaudes C, Bright MG (2024). Denoising task-correlated head motion from motor-task fMRI data with multi-echo ICA. Imaging Neurosci 2:10.1162/imag_a_00057. doi: 10.1162/imag_a_00057.

Reddy NA, Clements RG, Brooks JCW, Bright MG (2024). Simultaneous cortical, subcortical, and brainstem mapping of sensory activation. Cereb Cortex 34(6):bhae273. doi: 10.1093/cercor/bhae273.

Richter D, Ekman M, de Lange FP (2018). Suppressed Sensory Response to Predictable Object Stimuli throughout the Ventral Visual Stream. J Neurosci 38(34):7452-7461. doi:10.1523/JNEUROSCI.3421-17.2018. Epub 2018 Jul 20. PMID: 30030402; PMCID: PMC6596138.

Russ BE, Leopold DA (2015). Functional MRI mapping of dynamic visual features during natural viewing in the macaque. Neuroimage 109:84-94.

Sadagopan S, Temiz-Karayol NZ, Voss HU (2015). High-field functional magnetic resonance imaging of vocalization processing in marmosets. Sci Rep. 2015 Jun 19;5:10950. doi: 10.1038/srep10950. PMID: 26091254; PMCID: PMC4473644.

Strigo IA, Guerra SG, Torrisi S, Murphy E, Toor T, Goldman V, Alter BJ, Vu AT, Hecht R, Lotz J, Simmons AN, Mehling WE (2024). Enhancing chronic low back pain management: an initial neuroimaging study of a mobile interoceptive attention training. Front Pain Res 5:1408027. doi: 10.3389/fpain.2024.1408027. PMID: 39403233; PMCID: PMC11471628.

Thirion B, Varoquaux G, Grisel O, Poupon C, Pinel P (2014). Principal component regression predicts functional responses across individuals. Med Image Comput Comput Assist Interv. 17(Pt 2):741-8. doi: 10.1007/978-3-319-10470-6_92. PMID: 25485446.

van den Bosch R, Lambregts B, Määttä J, Hofmans L, Papadopetraki D, Westbrook A, Verkes RJ, Booij J, Cools R (2022). Striatal dopamine dissociates methylphenidate effects on value-based versus surprise-based reversal learning. Nat Commun 13(1):4962. doi: 10.1038/s41467-022-32679-1. PMID: 36002446; PMCID: PMC9402573.

van den Bosch R, Hezemans FH, Määttä JI, Hofmans L, Papadopetraki D, Verkes RJ, Marquand AF, Booij J, Cools R (2023). Evidence for absence of links between striatal dopamine synthesis capacity and working memory capacity, spontaneous eye-blink rate, and trait impulsivity. Elife 12:e83161. doi: 10.7554/eLife.83161. PMID: 37083626; PMCID: PMC10162803.

van Lieshout LLF, Vandenbroucke ARE, Müller NCJ, Cools R, de Lange FP (2018). Induction and Relief of Curiosity Elicit Parietal and Frontal Activity. J Neurosci 38(10):2579-2588. doi:10.1523/JNEUROSCI.2816-17.2018. Epub 2018 Feb 8. PMID: 29439166; PMCID: PMC6705901.

Zandbelt B (2017). Slice Display. figshare. 10.6084/m9.figshare.4742866

Ziontz J, Bilgel M, Shafer AT, Moghekar A, Elkins W, Helphrey J, Gomez G, June D, McDonald MA, Dannals RF, Azad BB, Ferrucci L, Wong DF, Resnick SM (2019). Tau pathology in cognitively normal older adults. Alzheimers Dement (Amst) 11:637-645. doi: 10.1016/j.dadm.2019.07.007. PMID: 31517026; PMCID: PMC6732758.
